# Short-term *Cudrania tricuspidata* fruit vinegar administration attenuates obesity in high-fat diet-fed mice by improving fat accumulation and metabolic parameters

**DOI:** 10.1038/s41598-020-78166-9

**Published:** 2020-12-03

**Authors:** Jun-Hui Choi, Myung-Kon Kim, Soo-Hwan Yeo, Seung Kim

**Affiliations:** 1 Department of Food Science and Biotechnology, Gwangju University, Gwangju, 503-703 Republic of Korea; 2 Department of Food Science and Technology, Chonbuk National University, Iksan, 570-752 Republic of Korea; 3 Fermented Processing Food Science Division, Department of Agrofood Resource, National Academy of Agricultural Science, RDA, Wanju, 55365 Republic of Korea

**Keywords:** Biochemistry, Molecular biology, Biomarkers

## Abstract

Previous studies have suggested that vinegar intake can help to reduce body fat and hyperglycemia. Therefore, this study aimed to evaluate the anti-obesity efficacy of vinegar fermented using *Cudrania tricuspidata* fruits (CTFV) and its main phenolic constituents and to analyze its molecular mechanism and changes in obesity-related metabolizing enzymatic activities. We found that HFD significantly caused hepatic steatosis; increases in body fats, feed efficiency, liver mass, lipids, insulin, oxidative parameters, cardiovascular-associated risk indices, lipase and α-amylase activities, whereas CTFV efficaciously attenuated HFD-induced oxidant stress, fat accumulation, obesity-related enzymatic activity, and the activation or reduction of obesity-related molecular reactions via improving metabolic parameters including phosphorylated insulin receptor substrate 1, protein tyrosine phosphatase 1B, phosphorylated phosphoinositide 3-kinase/protein kinase B, phosphorylated mitogen-activated protein kinases, sterol regulatory element-binding protein 1c, CCAAT/enhancer-binding protein, and fatty acid synthase; and decreases in adiponectin receptor 1, leptin receptor, adenosine monophosphate-activated protein kinase, acetyl-CoA carboxylase, and peroxisome proliferator-activated receptor, subsequently ameliorating HFD-induced obesity. Therefore, CTFV might provide a functional food resource or nutraceutical product for reducing body fat accumulation.

## Introduction

The global obese population is on a constant rise due to a number of factors including a calorific surplus and environmental determinants^1^, and the global studies are actively conducted on the management and treatment of obesity classified as a disease^2,3^. An estimated 650 million adults, 13% of the world’s adult population were obese in 2016, and 19.7% of the world’s population will be obese by the year of 2030^3^. In the case of the Republic of Korea, the rate of adult obesity has been on a steady rise to 35.5% in 2016, compared with 25.8% in 1988 and it's expected to increase rapidly by 2030^4^. Recently, as it is recognized that the risk of obesity is high, there is a growing interest in obesity treatment and weight control, and various medicines and herbal medicines for obesity treatment are being developed^5,6^. Various inhibitor drugs for the treatment of obesity are specifically effective in acting on targeted metabolic diseases, however, these drugs often have side effects such as insomnia, headache, palpitation, irritability, agitation, nervousness, stroke, heart attack, flatulence, diarrhea, abdominal pain, bloating, nausea, dyspepsia, arthralgia, dizziness, constipation, and dry mouth^5^.

Vinegar has long been used in various foods, including medicinal foods; in modern times, it serves as a flavor source for foods in many countries worldwide. The use of such vinegars is expanding due to increased numbers of obese people^7–10^, the entry into an aging society, an increased national income level^11^, and an improved quality of life in the Republic of Korea^12,13^. The commercialization and demand of food production using vinegar for effective fat reduction has steadily increased since, among vinegars, fermented pomegranate fruit vinegar (PFV) was approved by the Korean Food and Drug Administration (KFDA) as a functional ingredient to help reduce body fat for the first time^14,15^. Several previous studies have shown that vinegar intake reduced the risk factors for high cholesterol and atherosclerosis in rabbits^16^ and reduced food intake, body weight, and lipid levels in an obese mice model^17^; furthermore, the acetic acid contained in vinegar affected blood lipid levels and reduced body weight^18^, and vinegar supplementation reduced glucose levels and postprandial hyperglycemia in clinical study^19,20^. Recently, vinegar has been reported to show various biological effects, including antimicrobial, antitumor, antioxidant, anti-hepatic fibrosis, and anti-kidney stone recurrence activities as well as effects in reducing body fat and glucose levels^21–25^. To enhance the efficacy of vinegar, vinegars utilizing some fruits with beneficial health effects have been studied and produced; in fact, it was reported that various fruit types including black raspberries, *Schizandra chinensis* fruit, *Vitis coignetiae* fruit, apples, and blueberries were used to develop fruit vinegar^13,26,27^.

*Cudrania tricuspidata*, which is distributed mostly in East Asia and belongs to the family Morcophyta, is used as a food or medicinal plant. In particular, the fruit of *C. tricuspidata* is generally consumed as dried or fresh fruits, alcoholic beverages, and jams; additionally, the fruit contain various bio-active substances including flavone, isoflavonoid, and phenolic compounds^28^. In recent years, studies regarding the anti-obesity effects by *C. tricuspidata* fruits and its ingredients have been continuously reported^28–30^. However, there is little information regarding the mechanism and in vitro and in vivo efficacy of *C. tricuspidata* fruit vinegar (CTFV), and it has yet to be utilized as an ingredient for fermented functional foods. Therefore, the present study was designed to analyze the anti-obesity effects of CTFV and its polyphenolics and to compare the efficacies of CTFV, PFV, and fenofibrate in high-fat diet (HFD)-fed obese mice.

## Methods

### Chemicals, reagents, and antibodies

Pancreatic lipase, lipoprotein lipase, β-glucosidase, α-amylase, phosphodiesterase type-IV, citrate synthase, alkaline phosphatase, *p*-nitrophenyl butyrate, *p*-nitrophenyl-β-D-glucopyranoside, *p*-nitrophenyl phenylphosphonate (*p*-NPPPh), acetyl-coenzyme A (acetyl-CoA), 5,5′-dithiobis(2-nitrobenzoic acid) (DTNB), oxaloacetic acid (OAA), *p*-nitrophenyl phosphate (*p*-NPP), starch, trizma base, gallic acid, protocatechuic acid, chlorogenic acid, *p*-hydroxybenzoic acid, caffeic acid, isovanillic acid, rutin, *p*-coumaric acid, ferulic acid, taxifolin, coumaric acid, rosmarinic acid, quecertin, cinnamic acid, gastrodin, *p*-hydroxybenzyl alcohol, ethylenediaminetetraacetic acid (EDTA), dimethyl sulfoxide (DMSO), fenofibrate, hematoxylin and eosin, and parishin A, B, C, and E were purchased from Sigma-Aldrich (St. Louis, MO, USA). Dulbecco’s modified Eagle’s medium (DMEM), fetal bovine serum (FBS), penicillin, and streptomycin were purchased from Invitrogen (Carlsbad, CA, USA). PageRuler TM Plus Prestained protein ladder was purchased from Thermo Scientific (Rockford, IL, USA). Primary antibodies against adiponectin receptor 1 (adipoR1), phosphorylated leptin receptor (pOBR), OBR, phosphorylated insulin receptor substrate 1 (pIRS1), IRS1, phosphorylated extracellular-signal-regulated kinase (pERK), phosphorylated c-Jun N-terminal protein kinase (pJNK), phosphorylated P38 (pP38), phosphorylated phosphoinositide 3-kinase (pPI3K), PI3K, phosphorylated protein kinase B (pAKT), AKT, phosphorylated acetyl-CoA carboxylase (pACC), ACC, phosphorylated adenosine monophosphate-activated protein kinase (pAMPK), AMPK, glucose transporter 4 (GLUT4), sterol regulatory element-binding protein 1c (SREBP1C), peroxisome proliferator-activated receptor α (PPARα), peroxisome proliferator-activated receptor γ (PPARγ), CCAAT/enhancer-binding protein α (CEBPα), CCAAT/enhancer-binding protein β (CEBPβ), fatty acid synthase (FAS), and protein tyrosine phosphatase 1B (PTP1B) were purchased from Santa Cruz Biotechnology, Inc. (Santa Cruz, CA, USA). Other reagents were commercially available and of special grade.

### Wine and vinegar preparation

Fresh *C. tricuspidata* fruits were collected from plants cultivated in a farm located in Milyang district, Gyeongsangnam-do, Republic of Korea and immediately stored at − 20 °C until use. Before preparation of the wine, the frozen fruit was homogenized with tap water at a 1:2 ratio using a multipurpose high-performance hand blender (Lacuzin, China). The sets of mixture were raised to 24°Brix by adding sugar and sterilized for 90 min at 85 °C. The yeast strain (*Saccharomyces cerevisiae* Fermivin) was cultivated by inoculating into malt medium (12°Brix) and incubating at 25–27 °C for 5 days under shaking at 120 rpm. The fermentation process was then initiated by inoculating 6 L of the homogenized fruit slurry in a 10 L glass jar with 5% of the yeast culture. The fermentation jar was then incubated at 26 °C for 10 days with manual shaking twice daily. At the end of fermentation, the two wine phases (liquid and solid) were separated by centrifugation at 9000×*g* for 15 min, and the liquid phase was filtered through a 110 mm filter (Whatman filter paper no. 2). The *C. tricuspidata* fruit wine was then sterilized for 30 min at 85 °C. To prepare the vinegar, equal volumes of the *C. tricuspidata* fruit wine and traditional starter vinegar (acidity 7.2; described previously^31^) were mixed and incubated at 30 °C for 60 days. During incubation, fresh wine feeding (half of the volume of the vinegar preparation) and acidity measurements were conducted every twelve days.

### HPLC analysis

HPLC analysis was performed by the previously described method^32^ using an HPLC system (Waters, Milford, MA, USA) equipped with a 2690 separation module and Waters 996 DAD with a ZORBAX Eclipse XDB-C18 column (250 mm × 4.6 mm, 5 μm; Agilent Technologies, Inc., Santa Clara, CA, USA). For analysis of phenolic acids and flavonoids, the mobile phase comprised 0.1% formic acid in 10% acetonitrile (solvent A) and 0.1% formic acid in 90% acetonitrile (solvent B). The mobile phase ratio was maintained at A:B 100:0 (0–5 min), 100:0 (5–10 min), 88:12 (10–40 min), 30:70 (40–45 min), and 100:0 (45–50 min) at a flow rate of 0.8 mL/min. The UV–Vis absorption spectra were recorded between 200–400 nm during HPLC analysis, and the quantification of individual compounds was based on peak areas at 280 nm.

HPLC analysis of parishin derivatives was performed by the previously described method^32^. The mobile phase comprised 0.1% formic acid in ionized water (solvent A) and 0.1% formic acid in methanol (solvent B). The mobile phase ratio was maintained at A:B 95:5 (0–5 min), 85:15 (5–10 min), 45:55 (10–25 min), and 95:10 (25–40 min) at a flow rate of 0.8 mL/min. The UV–Vis absorption spectra were recorded between 200–400 nm during HPLC analysis, and the quantification of individual compounds was based on peak areas at 220 nm.

Twenty phenolic compound standards were used for calibration curves: protocatechuic acid, chlorogenic acid, caffeic acid, isovanillic acid, rutin, *p*-coumaric acid, ferulic acid, taxifolin, gallic acid, *p*-hydroxybenzoic acid, coumaric acid, rosmarinic acid, quecertin, cinnamic acid, gastrodin, *p*-hydroxybenzyl alcohol, and parishin A, B, C, and E. The standard solutions (50, 100, 250, and 500 μg/mL) were prepared in DMSO. The main compounds from CTFV were identified based on the retention times of the standards and quantified by comparing their peak area intensities with those of standard curves.

### Pancreatic and lipoprotein lipase assay

The enzyme activity of pancreatic and lipoprotein lipase was measured by the previously described method^30^. The enzyme solutions were prepared by adding pancreatic lipase (10 mU) or lipoprotein lipase (10 mU) to 10 mM Tris-HCl (pH 6.8) containing 10 mM MOPS and 5 mM CaCl_2_ for a total volume of 10 μL. The pancreatic lipase enzymes were pretreated with the extract (10 μg) or each compound (10 μg) in a total volume of 20 μL for 10 min at room temperature (RT). After 10 min pretreatment, the mixtures were added to 80 μL of 3 mM *p*-nitrophenyl butyrate; then, the reaction was initiated and continued for 30 min at 37 °C. After incubation, the activity of the reaction mixtures was determined by reading the absorbance at 405 nm using a microplate reader (Molecular Devices, Sunnyvale, CA, USA). Furthermore, 1 mU enzymatic activity indicates the enzyme quantity that catalyzes the hydrolysis of 1.0 nmol *p*-nitrophenyl butyrate per min under assay conditions.

1 $$ {\text{Lipaseinhibitory}}\;{\text{effect}}\;(\% ) = \left[ {{{\left( {{\text{Absorbance}}_{{{\text{control}}\;{\text{without}}\;{\text{sample}}}} {-}{\text{Absorbance}}_{{{\text{control}}\;{\text{with}}\;{\text{sample}}}} } \right)} \mathord{\left/ {\vphantom {{\left( {{\text{Absorbance}}_{{{\text{control}}\;{\text{without}}\;{\text{sample}}}} {-}{\text{Absorbance}}_{{{\text{control}}\;{\text{with}}\;{\text{sample}}}} } \right)} {{\text{Absorbance}}_{{{\text{control}}\;{\text{ without }}\;{\text{sample}}}} }}} \right. \kern-\nulldelimiterspace} {{\text{Absorbance}}_{{{\text{control}}\;{\text{ without }}\;{\text{sample}}}} }}} \right] \times 100 $$

### β-Glucosidase assay

The enzyme activity of β-glucosidase was determined by the previously described method, with slight modification^30^. The enzyme solution was prepared by adding β-glucosidase (0.1 U) to 10 mM phosphate-buffered saline (PBS) (pH 6.8) for a total volume of 10 μL. The enzyme was pretreated with 10 μL of the extract (10 μg) or each compound (10 μg) for 10 min at RT. After incubation, 80 μL of 3 mM *p*-nitrophenyl-β-D-glucopyranoside was added to the mixtures, and the reaction was initiated and continued for 10 min at 37 °C. After incubation, the activity of the reaction mixtures was measured by reading the absorbance at 405 nm using a microplate reader. The β-glucosidase inhibitory effect (%) was expressed as the percent difference from the residual activity, as shown in Eq. (1). Furthermore, 1 mU enzymatic activity is defined as the enzyme quantity required to release 1 nmol para-nitrophenol from *p*-nitrophenyl-β-D-glucopyranoside per min under assay conditions.

### α-Amylase assay

The α-amylase activity analysis was performed by the previously described method^33^. For use as a specific substrate, 0.3 g starch was dissolved in 1.5 mL of 0.4 M NaOH and heated for 10 min at 98 °C. After cooling in ice, the solution pH was adjusted to 7.0 using 2.0 M HCl in a total volume of 2 mL. The enzyme (10 mU) was pretreated with 10 μL of the extract (10 μg) or each compound (10 μg) in a total volume of 50 μL for 10 min at RT. After incubation, 50 μL of the substrate solution and 50 μL of the reaction solution or each compound were incubated at 35 °C for 30 min. The absorbance of α-amylase enzyme was measured at 580 nm. The α-amylase inhibitory effect (%) was expressed as the percent difference from the residual activity, as shown in Eq. (1). Furthermore, 1 U α-amylase is the enzyme quantity that releases 1.0 mg maltose from starch in 3 min at pH 6.9 at 20 °C.

### Phosphodiesterase IV assay

The enzyme solution was prepared by adding phosphodiesterase IV (2 mU) to 10 mM PBS (pH 6.8) for a total volume of 10 μL according to the previously described method^30^, and *p*-NPPPh was used as a specific substrate for phosphodiesterase IV. The substrate solution (10 mM) was prepared by dissolving the substrate in PBS. Phosphodiesterase IV was pretreated with 10 μL of the extract (10 μg) or each compound (10 μg) for 10 min; then, the reaction was initiated and continued for 10 min at 37 °C after adding 10 mM *p*-NPPPh at a total volume of 100 μL. After incubation, the amount of 4-nitrophenol released was determined by reading the absorbance at 405 nm using a microplate reader. The phosphodiesterase IV inhibitory activity (%) was expressed as the percent difference from the residual activity, as shown in Eq. (1). The enzyme activity was expressed in mU/min, and 1 mU was defined as the enzyme quantity required to hydrolyze 1 nmol *p*-NPPPh under assay conditions.

### Citrate synthase assay

The enzyme activity of citrate synthase was determined at 412 nm with a microplate reader by the previously described method, with slight modification^30^. Assays were performed at RT in a solution of 10 mM Tris-HCl buffer (pH 8.0), 0.2 mM acetyl-CoA, 0.3 mM OAA, and 0.1 mM DTNB at a volume of 100 μL. The enzyme (0.05 U) was pretreated with the extract (10 μg) or each compound (10 μg) for 10 min. The reaction was initiated by adding the enzyme (0.05 U) and analyzed by the reaction of CoA with DTNB for 30 min. After 30 min, the inhibitory effect of the extract or each compound was presented as the percent difference from the *V*_max_ (mU/min) of citrate synthase. Finally, 1 mU of activity was defined as the enzyme quantity required to generate 1 nmol CoA per min.

2 $$ {\text{Citrate}}\;{\text{synthaseinhibition}}\;{\text{effect}}\;(\% ) = \left[ {{{{1} - V_{{{\text{max}}\;{\text{control}}\;{\text{ with}}\;{\text{ sample}}}} } \mathord{\left/ {\vphantom {{{1} - V_{{{\text{max}}\;{\text{control}}\;{\text{ with}}\;{\text{ sample}}}} } {V_{{{\text{max}}\;{\text{control }}\;{\text{without }}\;{\text{sample}}}} }}} \right. \kern-\nulldelimiterspace} {V_{{{\text{max}}\;{\text{control }}\;{\text{without }}\;{\text{sample}}}} }}} \right] \times {1}00 $$

### Alkaline phosphatase assay

Alkaline phosphatase activity was determined by the previously described method, with slight modification^34^ using a *p*-NPP liquid substrate system at 405 nm. The enzyme (10 mU) was pretreated with the extract (10 μg) or each compound (10 μg) for 10 min and then treated with 10 mM *p*-NPP for 15 min at RT. After treatment, the activity of the reaction mixtures was measured by the absorbance values read at 405 nm with a microplate reader by monitoring the release of *p*-nitrophenol from *p*-NPP. One unit of alkaline phosphatase activity was defined as 1 μmol *p*-nitrophenol released per min. The alkaline phosphatase inhibitory effect (%) was expressed as the percent difference from the residual activity, as shown in Eq. (1).

### Cell culture

Cell culture was performed according to the method described^35^. HepG2, 3T3-L1, and Raw264.7 cells were obtained from the American Type Culture Collection (Manassas, VA, USA) and cultured separately in DMEM supplemented with 10% FBS, 100 U/mL penicillin, and 100 μg/mL streptomycin. The cells were incubated at 37 °C in humidified air (5% CO_2_, 95% air). The media were changed every 2 days. The extract was dissolved in saline solution. To examine possible toxic effects, HepG2, 3T3-L1, and Raw264.7 cells were treated with 30–1000 μg/mL CTFV for 24 h.

### Cell viability assay

Cell viability was determined using the 3-(4,5-dimethylthiazol-2-yl)-2,5-diphenyltetrazolium bromide (MTT) reduction assay according to the method described^35^. Cells were seeded into 96-well plates at a density of 1 × 10^4^ cells/well and incubated for 24 h before experimental treatments. After treatment with CTFV for the indicated times, MTT was added to each well at a final concentration of 0.5 mg/mL. After incubation at 37 °C for 4 h, the culture media containing MTT were carefully removed. Next, 100 μL dimethyl sulfoxide was added to each well for 10 min to dissolve the formazan crystals, and absorbance was measured at 570 nm using a microplate reader. Wells without cells were used as blanks and were subtracted from each sample as the background.

### Animals

Fifteen-week-old Male Imprinting Control Region (ICR) mice (40–50 g) were used for the high-fat diet (HFD)-fed obese mice model experiment. Four animals were housed per cage and maintained under controlled environmental conditions (22 ± 2 °C, 12 h light/12 h dark cycle). Feed (LabDiet 5L79; ORIENT BIO Inc., Seongnam, Korea) or an HFD with 60% fat (diet-induced obesity (DIO) Rodent Purified Diet w/60%, TestDiet 58Y1; ORIENT BIO Inc., Seongnam, Korea) and tap water were available ad libitum. The compositions of the administered diets are shown in Table 1. Efforts were made to minimize the animals’ suffering and reduce the number of animals used. All experimental procedures were performed in accordance with the National Institutes of Health Guide for the Care and Use of Laboratory Animals (NIH publication no. 80–23, revised 1996) and related ethical regulations of Gwangju University, and were approved by the Institutional Animal Care and Use Committee of Jeonnam Institute of Natural Resources Research, Jangheung, South Korea (2018-JINR1814).

**Table 1:** Composition of the experimental diets.

Composition	w/w				
	Control	DIO	Feno	PFV	CTFV
Nitrogen-free extract	59.5	37.5	37.5	37.5	37.5
Fat	4.5	34.9	34.9	34.9	34.9
Protein	20.1	23.6	23.6	23.6	23.6
Fiber	4.6	–	–	–	–
Ash	5.8	–	–	–	–
Mineral mixture	3.5	3	3	3	3
Vitamin mixture	1	1	1	1	1
Fenofibrate	–	–	0.16	–	–
PFV	–	–	–	0.4	–
CTFV	–	–	–	–	0.4
Protein calories	21.0	18.1	18.1	18.1	18.1
Fat calories	13.7	61.6	61.6	61.6	61.6
Carbohydrates calories	65.3	20.3	20.3	20.3	20.3
Energy (kcal/g)	4.04	4.65	4.65	4.91	4.91

The diets composition according to LabDiet 5L79 and TestDiet 58Y1. DIO, high fat diet-induced obese mice DIO group; Feno, Fenofibrate-treated obese mice group; PFV, pomegranates fruits vinegar-treated obese mice group; CTFV, *C. tricuspidata* fruits vinegar-treated obese mice group.

### In vivo HFD-fed obese mice model and treatment groups

The anti-obesity effect of CTFV was investigated in mice with HFD-induced obesity. The mice were fed an HFD for 50 days, and fenofibrate (Feno) (200 mg/kg/day), PFV (500 mg/kg/day), or CTFV (500 mg/kg/day) were administrated orally for 50 days. The mice were divided into 5 groups with 10 mice per group. Group 1 included normal control mice fed a normal pellet diet and treated with saline as a vehicle (Control group). Group 2 comprised the obese mice model fed the HFD and treated with saline as a vehicle (DIO group). Groups 3, 4, and 5 included the obese mice model treated with Feno (DIO + Feno group), PFV (DIO + PFV), or CTFV (DIO + CTFV), respectively. Food intakes and body weights were recorded at regular 5-day intervals. The body weight gain (g) was calculated as (final biomass − initial biomass)/50 days. The feed efficiency ratio was calculated as biomass gain (final biomass − initial biomass) per mass of feed consumed (total feed supplied—total remaining feed). At the end of the study, after 12 h fasted state the animals in each group were anesthetized using light ether and sacrificed, and several tissues of the liver, kidney, spleen, fats, and blood samples were collected for tissue or biochemical analysis. Serum was obtained from 2 mL of whole blood clotted in test tube and then centrifuged at 1500×*g* for 15 min. The serum samples were stored at − 70 °C for further experiments. For the measurement of enzymatic activities and oxidative parameters, 10% liver homogenates in cold solution containing 0.5 mM ethylene glycol bis(2-aminoethyl ether)-*N*,*N*,*N*′,*N*′-tetraacetic acid (EGTA), 3 mM MgCl_2_, 10 mM KH_2_PO_4_, 20 mM 4-(2-hydroxyethyl)-1-piperazineethanesulfonic acid (HEPES), 110 mM sucrose (pH 7.0) were immediately prepared using a homogenizer. The homogenates were centrifuged at 800×*g* and 4 °C for 5 min. Protein level was measured by bicinchoninic acid (BCA) assay using bovine serum albumin as the standard.

### Liver and fat tissue histology

The white adipose tissue (epididymal, perirenal, and mesenteric fat) of the fat pad and liver were removed in an overnight fasting state and stored at − 70 °C before use. After removal, the liver was perfused in 10% formalin, and the liver and white adipose tissue were fixed in 10% formalin for 24 h. The sections of white adipose tissue were stained with hematoxylin and eosin. The adipocyte size in each group was measured and expressed as a percentage. Fat accumulation in frozen tissue was evaluated histologically using Oil Red O, where the frozen tissue was processed using cryostat, fixed, and stained. The color intensity of Oil Red O staining in each group was analyzed using ImageJ software (National Institutes of Health, Bethesda, MD, USA) and expressed as a percentage. Liver and fat pads were viewed under a Leica DM500 microscope (Leica, Heerbrugg, Switzerland).

### Catalase assay

Catalase (CAT) activity was assayed according to the method described, with modifications^36^. A mixture of 50 mM sodium phosphate buffer (pH 7.4), 1 μM H_2_O_2_, and serum or tissue homogenate formed a final volume of 1 mL, and the decrease in absorbance was measured at 240 nm over 10 min. One unit of CAT activity was defined as the enzyme quantity required to decompose 1 μM H_2_O_2_ in 1 min. Enzyme activity was expressed as U/g tissue protein.

### Superoxide dismutase assay

For the superoxide dismutase (SOD) assay, serum or tissue homogenate was mixed with 1 mM xanthine, 0.2 mM cytochrome, and 0.05 M potassium cyanide in 0.05 M potassium phosphate/0.1 mM EDTA buffer by the previously described method^37,38^. Xanthine oxidase was added to the reaction mixture, and the SOD activity was spectrophotometrically determined at 550 nm with a microplate reader as the inhibitory rate of cytochrome reduction by superoxide radical.

### Glutathione peroxidase assay

The activity of glutathione peroxidase (GPx) was determined by mixing serum or tissue homogenate with 1 mM EDTA, 100 mM GSH, and 5 mM NADPH by the previously described method^39^, followed by adding 1 U glutathione reductase in 0.1 M phosphate buffer (pH 7.0). After incubation for 3 min, 10 mM cumene hydroperoxide was added, and the oxidation of NADPH into NADP^+^ was monitored spectrophotometrically at 340 nm, in which 1 U GPx led to the formation of 1 μmol NADP^+^ per milligram of protein per min.

### Lipid peroxidation assay

The lipid peroxidation assay was carried out using the previously described method, with modifications^36^. First, 100 μL serum or tissue homogenate was mixed with a reaction mixture containing 30 μL of 0.1 M Tris–HCl buffer (pH 7.4) and 30 μL prooxidative solution (250 μmol/L FeSO_4_). The volume was adjusted to 300 μL with water before incubation at 37 °C for 1 h. The color reaction was developed by adding 300 μL 8.1% sodium dodecyl sulfate to the reaction mixture containing homogenate. Then, 500 μL acetic acid/HCl (pH 3.4) and 500 μL 0.8% TBA were added. This mixture was incubated at 100 °C for 1 h. The TBA-reactive species produced were measured at 532 nm, and the absorbance was compared with the standard curve prepared using malondialdehyde (MDA).

### Nitric oxide assay

Nitric oxide metabolite in serum or tissue homogenate was measured by the Griess reaction^38^. First, 100 μL of each sample was incubated with an equal volume of Griess reagent (1% sulfanilamide and 0.1% naphthyl ethylenediamine dihydrochloride in 2.5% polyphosphoric acid) for 10 min at RT. Then, the absorbance was measured at 540 nm with a microplate reader. The nitrite concentration was determined from the absorbance at 540 nm using sodium nitrite as the standard.

### In vivo metabolizing enzymes assay

In vivo enzymatic activities in serums and livers obtained from each group were evaluated by the enzyme assays including pancreatic lipase, lipoprotein lipase, β-glucosidase, α-amylase, phosphodiesterase IV, citrate synthase, and alkaline phosphatase with modifications^30,33,34^. The serum or liver homogenate were treated with each specific substrate, then, the absorbances of each reaction solution were measured at 405, 412, or 580 nm with a microplate reader. The absorbances were compared with the standard, and the enzymatic activities were expressed in U/mg protein.

### Biochemical analysis

The levels of triacylglycerol (TG) and total protein (TP) in the liver tissue extract and levels of total cholesterol (TC), high-density lipoprotein cholesterol (HDL), TG, aspartate aminotransferase (AST), alanine aminotransferase (ALT), glucose, TP, and albumin in serum were determined using a Vitros 250 chemistry system (Ortho Clinical Diagnostics, Raritan, NJ, USA). The low-density lipoprotein cholesterol (LDL) and very LDL (VLDL) levels were calculated using the following formula developed by Friedewald’s formula^40^: LDL = TC − (HDL − TG/5); VLDL = TG/5. The ratio of HDL and TC (HTR) was calculated as [HTR = HDL/TC]. Serum insulin, leptin, and adiponectin levels were determined using ELISA assay for mouse insulin (Cat. No. EZRMI), mouse leptin (Cat. No. EZML), and mouse adiponectin (Cat. No. EZMADP) (Sigma-Aldrich, St. Louis, MO, USA). The homeostasis model assessment of insulin resistance (HOMA-IR) index was calculated as [fasting serum glucose × fasting serum insulin/22.5] to assess insulin resistance^41^. The atherogenic index (AI, TG/HDL), atherogenic coefficient (AC, [TC − HDL]/HDL), cardiac risk ratio (CRR, TC/HDL), and coronary artery index (CAI, LDL/HDL) were determined using the equations developed by Ikewuchi & Ikewuchi^42^.

### Immunoblotting

Immunoblotting analysis was performed according to the method described^35^. The collected liver tissues were homogenized in normal saline and centrifuged at 10,000×*g* for 15 min at 4 °C. The protein concentrations were determined by BCA assay. Equal amounts of each tissue extract were separated by 12% sodium dodecyl sulfate–polyacrylamide gel electrophoresis and transferred to polyvinylidene difluoride membranes. After blocking in 5% non-fat dry milk with Tris-buffered saline/Tween-20 (TBST) buffer (10 mM Tris–HCl, 150 mM NaCl, and 0.1% Tween 20 [pH 7.5]) for 1 h at RT, the membranes were incubated with primary antibodies against adiponectin, leptin, adipoR1, pOBR, OBR, pIRS1, IRS1, pERK, pJNK, pP38, pPI3K, PI3K, AKT, pACC, ACC, pAMPK, AMPK, GLUT4, SREBP1C, PPARα, PPARγ, CEBPα, CEBPβ, FAS, and PTP1B (1:1000 dilution); pAKT (1:2000 dilution); and beta-actin (1:2500 dilution) for 1 h at RT. The membranes were washed three times in TBST buffer and incubated further with horseradish peroxidase-conjugated secondary antibodies for 1 h at RT. To reveal the reaction bands, the membranes were allowed to react with Western Blue Stabilized Substrate for WESTZOL (plus) Western Blot Detection System (Intron Biotechnology, Inc., Seongnam, Korea). The signals were detected by a MicroChemi instrument (DNR Bio-imaging Systems, Jerusalem, Israel).

### Statistical analysis

Statistical analysis was performed by the previously described method^30^ using SPSS 21 software (SPSS Inc., Chicago, IL, USA). The data collected and analyzed from this study were expressed as mean ± standard deviation (SD). The statistical significance of multiple group comparisons was assessed by one-way analysis of variance followed by a post-hoc Tukey’s test. *P*-values less than 0.05 were considered as statistically significant.

## Results

### CTFV production and physicochemical properties

The traditional fermentation method described in the text was applied to produce CTFV, whereas PFV (Daesang Corporation, Seoul, Korea) was brought from the local market to compare their efficacies. The CTF wine was fermented for 10 days and then incubated with a vinegar starter for 60 days. Various physical or chemical properties and alcohol and sugar contents may be changed during the alcoholic and acetic acid fermentation process. The pH values of juice, wine, and vinegar ranged from 3.73–5.44, as shown in Table 2. The highest pH was recorded in CTF juice, and it was reduced in wine and vinegar by pH 3.82 ± 0.04 and pH 3.30 ± 0.06, respectively. The mean sugar content of juice was found to be 24.0 ± 0.1°Brix, which decreased in wine (9.5 ± 0.2°Brix) and vinegar (20.0 ± 0.3°Brix). Table 6 shows that the total acidity of juice was 0.18 ± 0.01%, which increased in wine (0.58 ± 0.02) and vinegar (11.2 ± 0.28) after fermentation and incubation. Moreover, the mean alcohol concentration in wine was 13.0 ± 0.1%, while that in vinegar was 0.0%.

**Table 2:** The physicochemical properties of juice, wine, and vinegar from *C. tricuspidata* fruits.

	Fermentation day	pH	°Brix	Total acidity (%)	Alcohol (%)
Juice	0	5.44 ± 0.49	24.0 ± 0.1	0.18 ± 0.01	0.0
Wine	2	3.94 ± 0.05	23.0 ± 0.3	0.66 ± 0.05	2.2 ± 0.1
	4	3.73 ± 0.11	16.0 ± 0.2	0.79 ± 0.06	6.1 ± 0.1
	6	3.77 ± 0.10	12.0 ± 0.1	0.60 ± 0.03	11.8 ± 0.1
	8	3.81 ± 0.06	10.0 ± 0.1	0.55 ± 0.04	12.1 ± 0.2
	10	3.82 ± 0.04^#^	9.5 ± 0.2^#^	0.58 ± 0.02^#^	13.0 ± 0.1^#^
Vinegar	30	3.40 ± 0.05*	14.4 ± 0.4*	7.4 ± 0.23*	0.0
	60	3.30 ± 0.06*	20.0 ± 0.3*	11.2 ± 0.28*	0.0

Values are mean ± SD of 3 observations. ^#^*p* < 0.01, compared with the juice of *C. tricuspidata* fruits. *p < 0.01, compared with the wine of *C. tricuspidata* fruits.

### CTFV contains active ingredients, including polyphenolic and parishin derivatives

**Figure 1:**
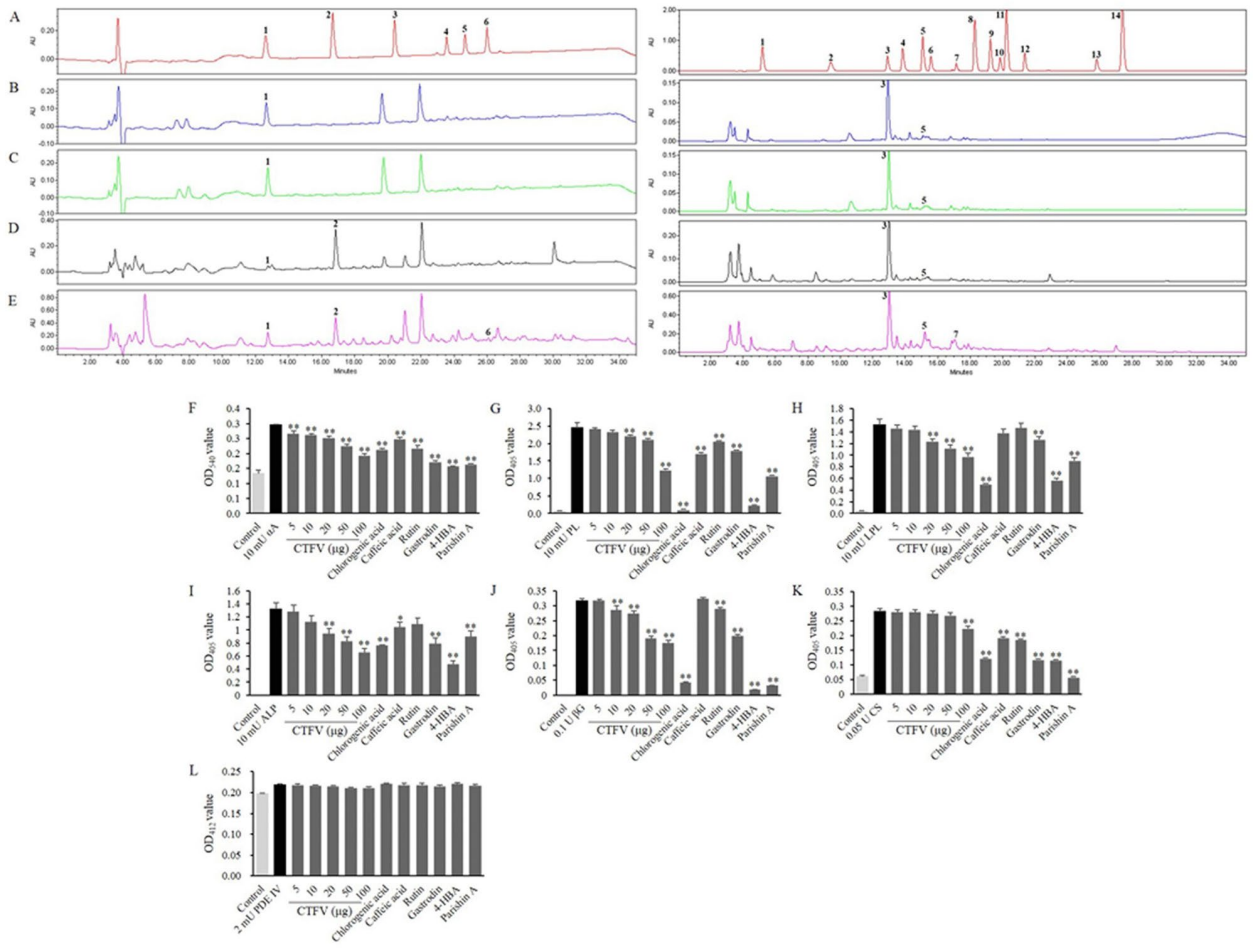
HPLC analysis (**A**–**E**) and inhibitory effects (**F**–**L**) in CTF and CTFV. (**A**) Mixture of authentic standards. (**B**) CTF juice before sterilization. (**C**) CTF juice after sterilization. (**D**) CTF wine. (**E**) CTF vinegar. *X*-axis is retention time in minutes and *Y*-axis is absorbance unit (AU). (1) gastrodin; (2) *p*-hydroxybenzyl alcohol; (3) parishin E; (4) parishin B; (5) parishin C; (6) Parishin A (upper-left panel). (1) gallic acid; (2) protocatechuic acid; (3) chlorogenic acid; (4) *p*-hydroxybenzoic acid; (5) caffeic acid; (6) isovanillic acid; (7) rutin; (8) *p*-coumaric acid; (9) ferulic acid; (10) taxifolin; (11) trans-coumaric acid; (12) rosmarinic acid; (13) quecertin; 14, trans-cinnamic acid (upper-right panel). After pretreatment with CTFV and the compounds, the mixtures were incubated for 10 or 30 min. After incubation, the activity of the reaction mixtures was determined by the absorbance values read at 405, 412, or 580 nm. The inhibitory effect was expressed as the percent difference from the residual activity by Eq. (1) (Inhibitory effect (%) = [(Absorbance _control without sample_ − Absorbance _control with sample_)/Absorbance _control without sample_] × 100) or Eq. (2) ([1 − *V*_max control with sample_/*V*_max control without sample_] × 100). Each value is the mean ± SD of triplicate measurements. *p < 0.05 and **p < 0.01, compared to non-treated each enzyme groups. αA, α-amylase; PL, pancreatic lipase; LPL, lipoprotein lipase; ALP, alkaline phosphatase; βG, β-glucosidase; CS, citrate synthase; PDE IV, phosphodiesterase IV.

In previous studies^28,32^, we found that CTF and its ferment contain various bio-active substances, including flavonoid, phenolic, and parishin derivatives, and exhibit the anti-oxidant and anti-obesity effects of the fruits. Hence, we examined the contents of polyphenolic compounds and parishin derivatives in CTFV using HPLC (Fig. 1A–E). Table 3 shows that the concentrations of gastrodin, *p*-hydroxybenzoic acid, and parishin A (as parishin derivatives) detected in CTFV were 274.3 ± 2.6, 120.2 ± 0.1, and 10.7 ± 0.4 μg/mL, respectively, at each peak compared to standards, and those of chlorogenic acid, caffeic acid, and rutin (as polyphenolics) were 331.9 ± 5.9, 46.2 ± 1.3, and 142.9 ± 4.2 μg/mL, respectively, compared to standards.

**Table 3:** Concentration of polyphenolic compounds, total phenol, and total flavonoid of *C. tricuspidata* fruits at different fermentation steps.

Fermentation step	Chlorogenic acid	Caffeic acid	Rutin	Gastrodin	*p*-Hydroxybenzoic acid	Parishin A	Total phenol	Total flavonoid
Concentration (μg/g dw)							Concentration (mg/g dw)	
CTF juice (before sterilization)	512.8 ± 17.9	29.8 ± 0.0	Trace	1197.4 ± 87.8	Trace	Trace	2.32 ± 0.11	0.43 ± 0.03
CTF juice (after sterilization)	540.9 ± 10.7	28.2 ± 0.0	Trace	1574.6 ± 14.6	Trace	–	2.33 ± 0.15	0.43 ± 0.03
CTF wine 13%	517.7 ± 8.3	Trace	Trace	105.9 ± 3.1	341.2 ± 1.2	–	2.62 ± 0.21	0.58 ± 0.04
CTF vinegar	1423.9 ± 25.3	198.2 ± 5.6	613.0 ± 18.0	1177.3 ± 11.2	515.7 ± 0.4	45.9 ± 1.7	3.55 ± 0.27	0.89 ± 0.07

The data are presented as means ± SD, *n* = 3. Total phenol content is expressed in mg GAE/g dw. Total flavonoid content is expressed in mg QUE/g dw. CTF, *C. tricuspidata* fruits; (−) compounds, not detected or not quantified; Trace, trace amounts.

### CTFV and its major compounds reduce metabolizing enzyme activities in vitro

We assayed the inhibition of metabolizing enzymes by determining their enzymatic activity after treatment with the vinegar product and its major substances. The highest concentration of CTFV exerted a reduction in the activities of α-amylase (Fig. 1F), pancreatic lipase (Fig. 1G), lipoprotein lipase (Fig. 1H), alkaline phosphatase (Fig. 1I), β-glucosidase (Fig. 1J), and citrate synthase (Fig. 1K). The inhibitory effects toward the activities of α-amylase, pancreatic lipase, lipoprotein lipase, alkaline phosphatase, and β-glucosidase increased proportionally to the dose at 5–100 μg. The enzymatic activity of phosphodiesterase IV did not differ significantly in any groups treated with CTFV or any compounds (Fig. 1L). All major compounds detected from CTFV inhibited α-amylase, pancreatic lipase, and citrate synthase. In particular, groups treated with chlorogenic acid, *p*-hydroxybenzoic acid, or parishin A showed strong reductions in pancreatic lipase, β-glucosidase, and citrate synthase activities.

### Effect of CTFV on cell viability in 3T3-L1, HepG2, and Raw263.7

**Figure 2:**
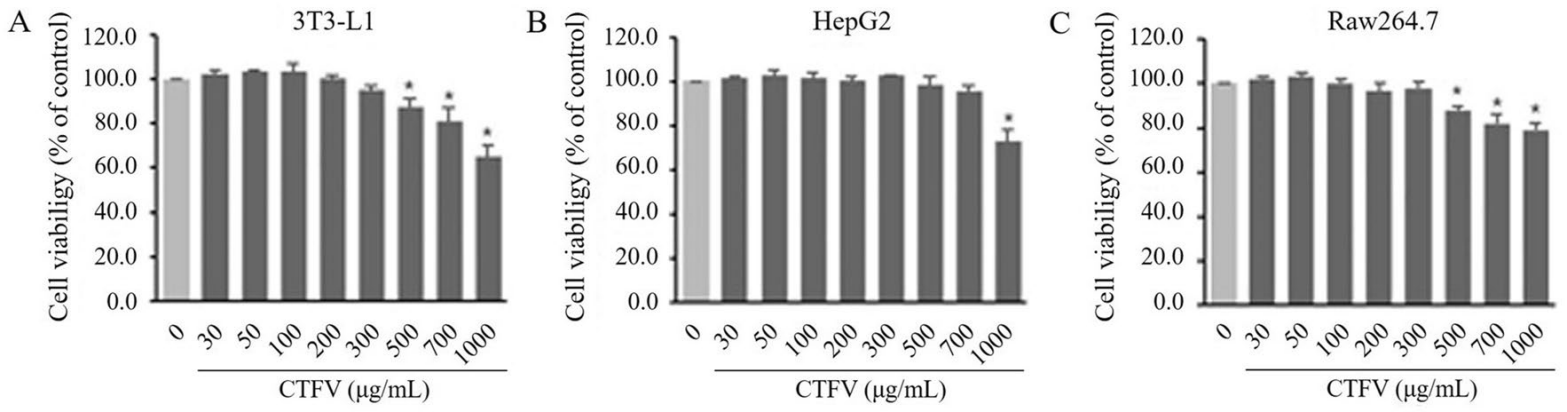
Assessment of cytotoxic effects. MTT assay showing the effect of CTFV on 3T3-L1 (**A**), HepG2 (**B**), and Raw264.7 cells (**C**) viability. Cells were incubated with the compound at different concentrations (0–1000 μg/mL) for 24 h and cell viability was analyzed by MTT reduction assay. Each value is the mean ± SD of triplicate measurements. *p < 0.01, compared with non-treated group.

The effect of CTFV on cell viability was tested by MTT assay of three cell lines: 3T3-L1, HepG2, and Raw263.7. Each cell line was treated with several concentrations of 30, 50, 100, 200, 300, 500, 700, and 1000 μg/mL CTFV for 48 h. Although CTFV treatment up to 300 μg/mL showed no effects on the viability of the three lines, 500–1000 μg CTFV decreased the viability of 3T3-L1 (Fig. 2A), HepG2 (Fig. 2B), and Raw264.7 (Fig. 2C).

### CTFV reduces body weight gain and decreases feed efficiency of HFD-fed obese mice model

**Figure 3:**
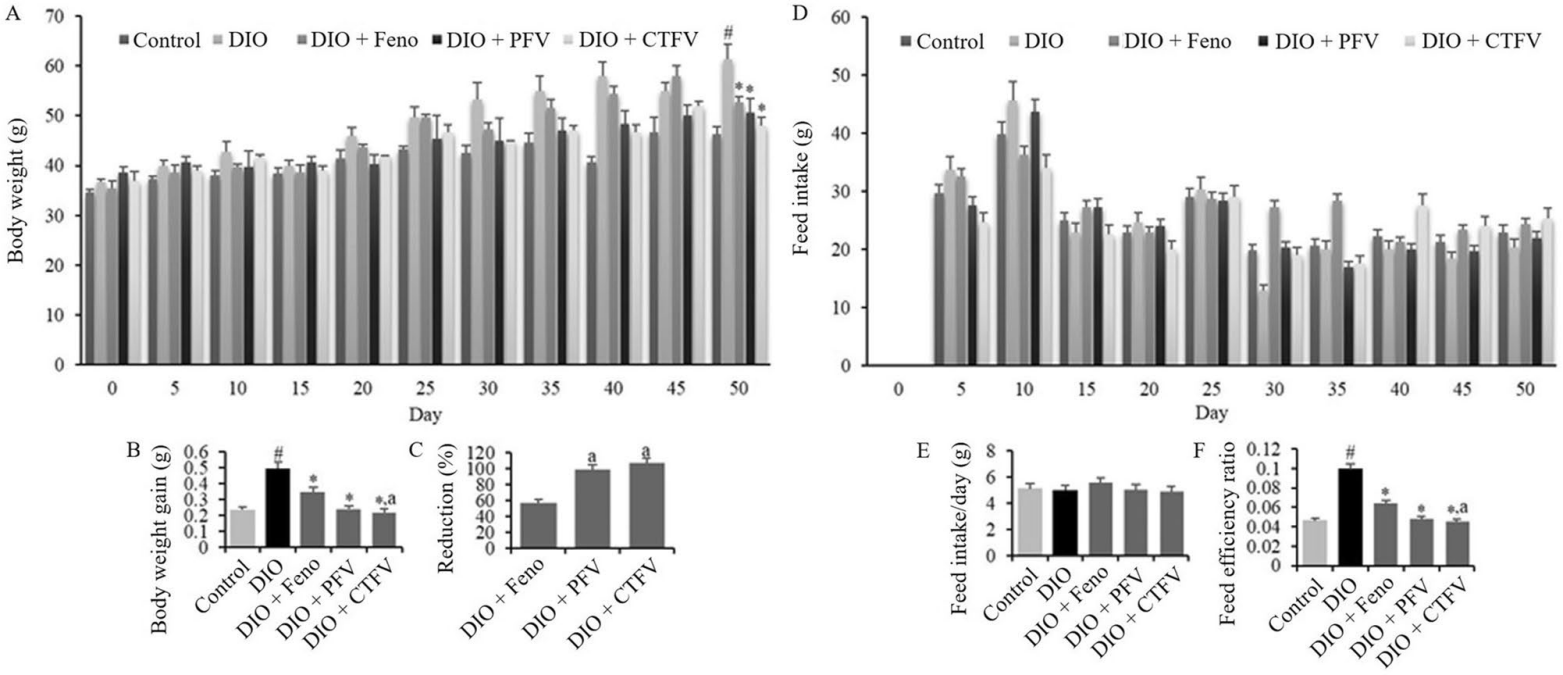
Effects of CTFV, PFV, and fenofibrate on body weight (**A**), body weight gain (**B**), reduction (%) (**C**), feed intake (**D**,**E**), and feed efficiency (**F**) in high fat-diet (HFD)-induced obese mice. Each value is the mean ± SD (n = 10). ^#^*p* < 0.01, compared with Control group, *p < 0.01, compared with DIO group, ^a^*p* < 0.01, compared with DIO + Feno group. Control, non-induced normal group; DIO, diet-induced obesity model group; DIO + Feno, fenofibrate-treated DIO group, DIO + PFV, pomegranate fruit vinegar (PFV)-treated DIO group; DIO + CTFV, *C. tricuspidata* fruit vinegar (CTFV)-treated DIO group.

To determine whether CTFV administration decreases the increase in body weight and feed efficiency in HFD-fed obese mice as a DIO model, body weight and food intake changes were recorded every fifth day. To the obese mice model, we administered CTFV, PFV as a positive control of a functional ingredient approved by the KFDA for fat reduction, or Feno as a second positive control of a lipid-regulating drug from the beginning of the experiment. As the DIO group, mice fed an HFD (TestDiet 58Y1 with 60% fat) for 50 days exhibited increased body weight and daily body weight gain compared to those of the control group, which was fed a normal diet (LabDiet 5L79) (Fig. 3A,B). The CTFV, PFV, and Feno groups displayed significant differences in the reduction in body weight gain compared with the DIO group; additionally, this reduction in the CTFV group was greater than that in the Feno group (Fig. 3C). While there was no significant difference between mean feed intakes (Fig. 3D,E), the HFD resulted in the increase of feed efficiency ratio compared with the Control group (Fig. 3F). CTFV administration reduced the feed efficiency ratio by 0.045 ± 0.003, respectively, compared with the DIO group (0.099 ± 0.006).

### Effect of CTFV on the mass of liver, kidney, spleen, and fat tissues

Table 4 shows the effects of CTFV, PFV, or Feno-administered groups on the weight of liver, kidney, spleen, and fat tissues. The final liver mass of HFD-fed mice was greater than that of the control group, whereas there were no significant changes in kidney and spleen masses. The HFD increased the weight of epididymal, perirenal, and mesenteric fats. However, CTFV administration reduced the mass gain of the liver and several fats, and the PFV and Feno groups showed a reduction in fat mass gain compared with the DIO group.

**Table 4:** Effect on liver, kidney, spleen, and fats tissue weights in high fat diet‑fed obese mice model.

Parameters	Control	DIO	DIO + Feno	DIO + PFV	DIO + CTFV
Liver	2.03 ± 0.12	3.40 ± 0.86^#^	2.10 ± 0.01	2.89 ± 0.75	1.91 ± 0.22*
Kidney	0.44 ± 0.02	0.49 ± 0.06	0.40 ± 0.04	0.44 ± 0.06	0.39 ± 0.05
Spleen	0.12 ± 0.01	0.14 ± 0.03	0.10 ± 0.02	0.13 ± 0.02	0.14 ± 0.02
Epididymal fat	0.61 ± 0.10	1.98 ± 0.30^##^	1.56 ± 0.18	1.20 ± 0.45*	1.26 ± 0.11*
Perirenal fat	0.05 ± 0.01	0.22 ± 0.03^##^	0.17 ± 0.01*	0.13 ± 0.02**	0.14 ± 0.01**
Mesenteric fat	0.13 ± 0.01	0.41 ± 0.06^##^	0.34 ± 0.01	0.32 ± 0.03*	0.27 ± 0.02**

The data are presented as means ± SD, *n* = 10. One-way ANOVA followed by the post hoc Tukey test. ^#^*p* < 0.05 and ^##^*p* < 0.01, compared to Control group. *p < 0.05 and **p < 0.01, compared to DIO group. Feno, fenofibrate; PFV, pomegranates fruits vinegar; CTFV, *C. tricuspidata* fruits vinegar.

### CTFV reduces hepatic fat and adipocyte growth in obese mice

**Figure 4:**
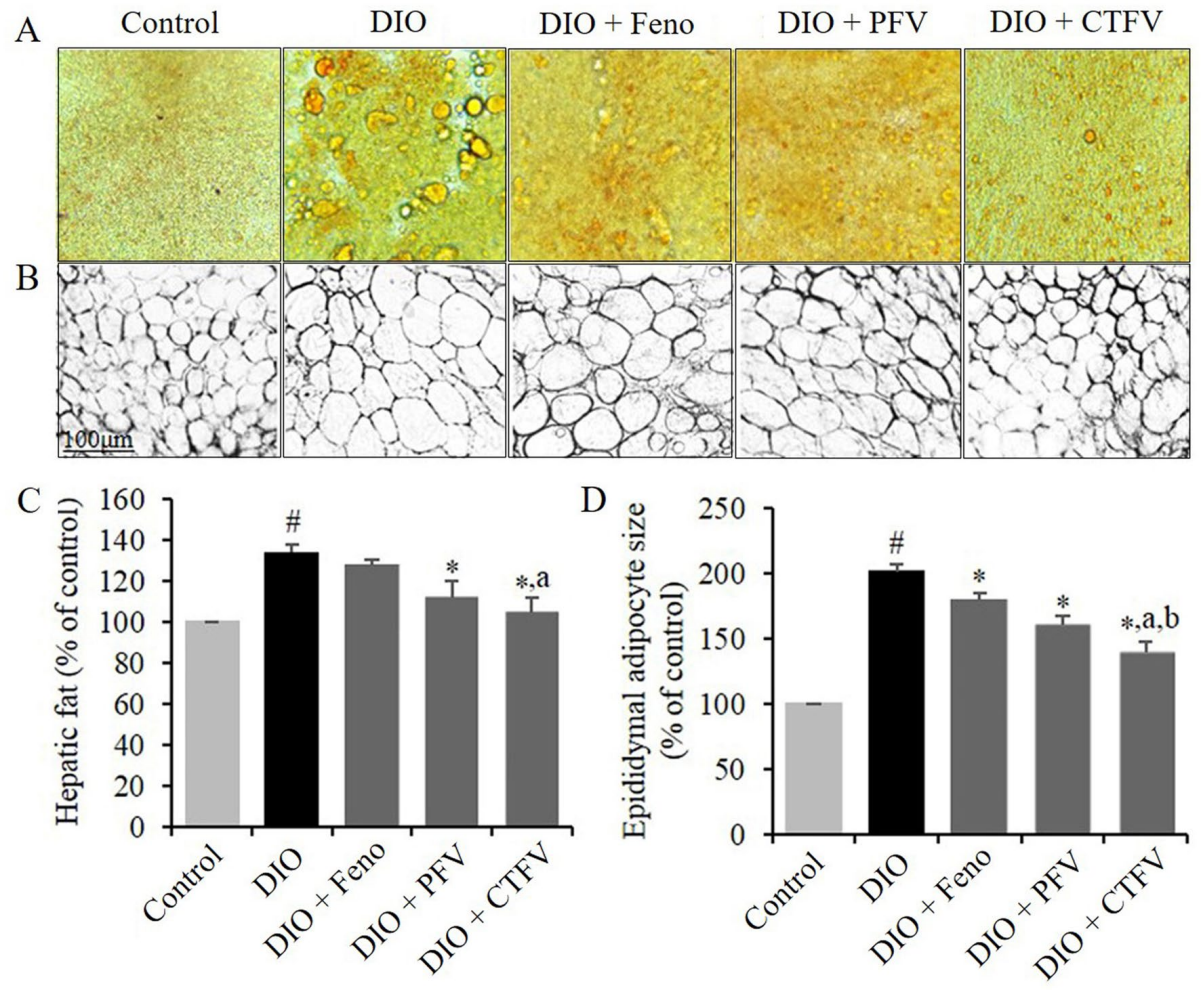
Representative histopathological analysis of the livers (**A**) and adipocyte tissues (**B**). Effects of CTFV, PFV, and fenofibrate on hepatic fat (**C**), and size of epididymal adipose tissue (**D**) in the obese mice were analyzed with H&E, microscope, and ImageJ. Each value is the mean ± SD of triplicate measurements. ^#^*p* < 0.01, compared with Control group, *p < 0.01, compared with DIO group, ^a^*p* < 0.01, compared with DIO + Feno group, ^b^*p* < 0.05, compared with DIO + PFV group. Control, non-induced normal group; DIO, diet-induced obesity model group; DIO + Feno, fenofibrate-treated DIO group, DIO + PFV, pomegranate fruit vinegar (PFV)-treated DIO group; DIO + CTFV, *C. tricuspidata* fruit vinegar (CTFV)-treated DIO group.

To investigate the effects of CTFV, PFV, or Feno administration on hepatic fat accumulation and adipocyte expansion, we analyzed the hepatic fat density and mean adipocyte size in each group. As shown in Fig. 4A,B, HFD accelerated a rise in hepatic fat accumulation (133.8 ± 3.6%) and mean adipocyte size (2.90 μm^2^ × 10^3^), compared with those (100% or 1.43 μm^2^ × 10^3^) of the Control group. The CTFV-administered group had the lowest mean level of fat density (104.2 ± 7.8%) (Fig. 4C) and smallest mean adipocyte size (1.98 μm^2^ × 10^3^) (Fig. 4D), compared with the PFV (111.9 ± 8.2% or 2.28 μm^2^ × 10^3^), and Feno groups (127.0 ± 3.5% or 2.55 μm^2^ × 10^3^), respectively.

### Effect of CTFV on oxidative stress parameters in serum

Serum oxidative stress parameters including antioxidant enzymes after CTFV, PFV, and Feno administration in obese mice were assessed due to the crucial role of body parts such as blood and liver on oxidative metabolism. As shown in Table 5, the DIO group revealed decreased activity levels of CAT, SOD, and GPx and increased serum levels of MDA and NOx compared with the Control group. However, CTFV or PFV administration upregulated the mean enzymatic activities of CAT and SOD and reduced lipid oxidation and its product. There were no significant changes in the levels of CAT, SOD, GPx, and NOx between each of the Feno, PFV, and CTFV groups.

**Table 5:** Effects on oxidative stress parameters in serum from high-fat diet-fed obese mice model.

Parameters	Control	DIO	DIO + Feno	DIO + PFV	DIO + CTFV
CAT	151.73 ± 6.21	135.13 ± 4.59^#^	137.74 ± 5.64	144.65 ± 4.13	143.99 ± 3.08
SOD	92.55 ± 6.48	76.55 ± 5.36	77.08 ± 6.40	80.22 ± 6.15	83.41 ± 5.84
GPx	13.89 ± 1.58	11.87 ± 0.89	12.56 ± 1.07	12.15 ± 0.86	11.87 ± 1.21
MDA	1.43 ± 0.11	3.28 ± 0.13^##^	2.19 ± 0.15*	1.75 ± 0.12*	1.92 ± 0.13*
NOx	9.22 ± 0.65	10.72 ± 0.24^#^	10.69 ± 0.48	10.57 ± 0.40	10.51 ± 0.36

The data are presented as means ± SD, *n* = 10. One-way ANOVA followed by the post hoc Tukey test. ^#^*p* < 0.05 and ^##^*p* < 0.01, compared to Control group. *p < 0.01, compared to DIO group. Feno, fenofibrate; PFV, pomegranates fruits vinegar; CTFV, *C. tricuspidata* fruits vinegar; CAT, catalase (U/mg protein); SOD, superoxide dismutase (U/mg protein); GPx, glutathione peroxidase (mU/mg protein); MDA, malondialdehyde (nM/mL); NOx, nitrite (nM/mL).

### Effect of CTFV on lipid, total protein, and oxidative stress parameters in liver

As observed in Table 6, HDF leads to changes in hepatic triacylglycerol and total protein. The liver TG and TP levels were higher in the DIO group compared with the Control group. In the Feno, PFV, and CTFV groups, a reduction in liver TG was confirmed; particularly, CTFV administration showed a powerful liver TG-reducing effect compared with the DIO or Control groups.

**Table 6:** Effects on lipid, total protein, and oxidative stress parameters in liver from high-fat diet-fed obese mice model.

Parameters	Control	DIO	DIO + Feno	DIO + PFV	DIO + CTFV
Liver TG	0.41 ± 0.02	0.89 ± 0.07^##^	0.46 ± 0.05*	0.43 ± 0.09**	0.27 ± 0.07*
Liver TP	30.0 ± 2.5	42.9 ± 5.6^#^	35.1 ± 0.5	37.3 ± 1.7	40.8 ± 5.3
CAT	38.05 ± 1.52	26.32 ± 1.28^##^	27.04 ± 1.16	29.92 ± 1.20*	29.31 ± 1.17
SOD	0.73 ± 0.03	0.46 ± 0.02^#^	0.45 ± 0.02	0.48 ± 0.02	0.54 ± 0.03*
GPx	0.27 ± 0.02	0.15 ± 0.02^##^	0.19 ± 0.002	0.18 ± 0.008	0.16 ± 0.02
MDA	1.28 ± 0.05	1.66 ± 0.04^##^	1.51 ± 0.06*	1.40 ± 0.05**	1.43 ± 0.06**
NOS	1.97 ± 0.08	2.43 ± 0.07^##^	2.39 ± 0.05	2.31 ± 0.04	2.28 ± 0.05

The data are presented as means ± SD, *n* = 10. One-way ANOVA followed by the post hoc Tukey test. ^#^*p* < 0.05 and ^##^*p* < 0.01, compared to Control group. *p < 0.05 and **p < 0.01, compared to DIO group. Feno, fenofibrate; PFV, pomegranates fruits vinegar; CTFV, *C. tricuspidata* fruits vinegar; TG, triacylglycerol (mg/dL); TP, total protein (mg/dL); CAT, catalase (U/mg protein); SOD, superoxide dismutase (U/mg protein); GPx, glutathione peroxidase (U/mg protein); MDA, malondialdehyde (nM/mL); NOS, nitric oxide synthesis (nM/min/mg protein).

The CTFV group had mean increases in CAT, SOD, and GPx activities compared with those of the DIO group, but only the change in SOD showed significance (Table 6). Additionally, this group showed an inhibitory effect against lipid oxidation. In the PFV and Feno groups, significant changes in CAT or MDA levels were observed, while there were no differences in NOS and GPx between each of the Feno, PFV, and CTFV groups.

### Effect of CTFV on metabolizing enzyme activities in serum

**Figure 5:**
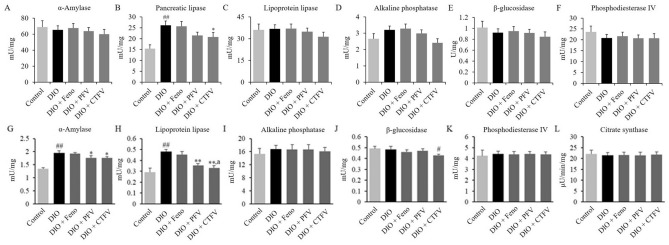
Effects of CTFV, PFV, and fenofibrate on α-amylase (**A**,**G**), pancreatic lipase (**B**), lipoprotein lipase (**C**,**H**), alkaline phosphatase (**D**,**I**), β-glucosidase (**E**,**J**), and phosphadesterase IV (**F**,**K**), and citrate synthase (**L**) in serum and liver from the obese mice. Each value is the mean ± SD of triplicate measurements. ^#^*p* < 0.05 and ^##^*p* < 0.01, compared with Control group, *p < 0.05 and **p < 0.01, compared with DIO group, ^a^*p* < 0.05 and ^aa^*p* < 0.01, compared with DIO + Feno group. Control, non-induced normal group; DIO, diet-induced obesity model group; DIO + Feno, fenofibrate-treated DIO group, DIO + PFV, pomegranate fruit vinegar (PFV)-treated DIO group; DIO + CTFV, *C. tricuspidata* fruit vinegar (CTFV)-treated DIO group.

The interruption of obesity-related enzymatic activities, including digestive and energy-metabolizing enzymes, might mediate their absorption and energy consumption. In the present study, the mean serum levels of pancreatic lipase, lipoprotein lipase, and alkaline phosphatase from the DIO group of obese mice were increased compared with those of the Control group (Fig. 5). Between the Control and DIO groups, pancreatic lipase showed a significant difference (Fig. 5B), but the other serum enzymes did not. In the serum from obese mice in the CTFV group, pancreatic lipase (20.77 ± 2.11 mU/mg protein) was reduced compared with that of DIO groups (26.18 ± 1.97 mU/mg protein) (Fig. 5B). The Feno and PFV groups showed no significant alteration in the enzymatic activity compared with the DIO group. These findings suggest that HFD causes the activation of pancreatic lipase and CTFV administration inhibits the activities of pancreatic lipase in serum.

### Effect of CTFV on metabolizing enzyme activities in liver

We further investigated changes in metabolizing enzyme activities in liver from obese mice administered CTFV, PFV, or Feno. As shown in Fig. 5, HFD caused mean increases in α-amylase and lipoprotein lipase activity in the liver. CTFV administration inhibited the activities of α-amylase (Fig. 5G), lipoprotein lipase (Fig. 5H), and β-glucosidase (Fig. 5J) compared with those of the DIO or Control groups, whereas there were no significant differences in alkaline phosphatase, β-glucosidase, phosphodiesterase IV, and citrate synthase in the CTFV group compared with the DIO group. In the PFV group alone, there were significant changes in α-amylase and lipoprotein lipase activities compared with those of the DIO group (Fig. 5G,H). These findings suggest that HFD causes the activation of α-amylase and lipoprotein lipase, and CTFV administration partially inhibits the increased activities of α-amylase and lipoprotein lipase in the liver.

### Effect of CTFV on serum biochemical parameters

HFD-induced obesity is known to be intimately related with lipid profiles, insulin resistance, adipokines, atherogenesis, and cardiac risk. Table 7 reveals the serum levels of the main biochemical parameters, including lipids, glucose, insulin, proteins, leptin, adiponectin, and some indices. Compared with the Control group, HFD feeding of DIO group mice for 50 days led to enhanced mean levels of all parameters and indices, except for adiponectin, which showed an average decrease; there were no significant differences in HDL, glucose, TP, albumin, leptin, and adiponectin values. CTFV administration reduced the values of lipid metabolic parameters including TC, TG, LDL, and VLDL, and increased the HDL mean value. The Feno group showed strong reductions in TC and LDL values; moreover, the PFV group had decreases in TG and LDL values. Serum insulin levels and insulin resistance of CTFV-administered obese mice were lower than those of the DIO group. Increased serum AST and ALT activities were reduced in the CTFV, PFV, and Feno groups, except for ALT in the Feno group.

**Table 7:** Effects on lipid levels, and related parameters in serum from high-fat diet-fed obese mice model.

Parameters	Control	DIO	DIO + Feno	DIO + PFV	DIO + CTFV
TC	100.1 ± 9.2	155.0 ± 6.3^#^	95.3 ± 5.0**	138.7 ± 7.3	128.3 ± 3.6**
TG	150.0 ± 9.4	302.0 ± 14.6^#^	98.2 ± 3.0**	227.7 ± 7.1**	89.7 ± 12.1**
HDL	108.3 ± 3.1	115.7 ± 14.4	109.0 ± 9.8	125.0 ± 8.7	128.0 ± 8.2
LDL	21.7 ± 5.2	99.7 ± 7.5^#^	5.9 ± 2.1**	59.2 ± 4.5**	18.3 ± 3.4**
VLDL	30.0 ± 9.9	60.4 ± 8.1^#^	19.6 ± 6.2**	45.5 ± 7.4	17.9 ± 2.4**
HTR	108.2 ± 3.1	74.6 ± 9.3^#^	114.4 ± 10.3**	90.2 ± 7.0	99.8 ± 6.4*
Glucose	215.3 ± 27.3	238.7 ± 23.4	190.3 ± 19.5	223.0 ± 22.5	193.3 ± 18.7
Insulin	0.043 ± 0.015	0.191 ± 0.064^#^	0.037 ± 0.010**	0.080 ± 0.015**	0.042 ± 0.013**
HOMA-IR	0.40 ± 0.09	2.09 ± 0.95^#^	0.32 ± 0.11**	0.79 ± 0.19*	0.35 ± 0.06**
AST	90.2 ± 4.4	168.5 ± 9.3^#^	122.1 ± 6.1**	110.6 ± 7.3**	113.3 ± 6.6**
ALT	28.2 ± 2.3	39.9 ± 3.2^#^	35.3 ± 2.8	31.4 ± 3.3*	30.3 ± 2.9*
TP	5.30 ± 0.60	5.33 ± 0.40	5.20 ± 0.44	5.37 ± 0.38	5.43 ± 0.55
Albumin	2.40 ± 0.30	2.67 ± 0.12	2.46 ± 0.32	2.70 ± 0.26	2.71 ± 0.30
Leptin	5.32 ± 0.88	12.59 ± 2.17^#^	11.13 ± 1.80	9.82 ± 1.35	8.94 ± 1.02
Adiponectin	6.10 ± 0.32	4.83 ± 0.29	4.95 ± 0.37	5.22 ± 0.24	5.53 ± 0.31
AI	1.38 ± 0.13	2.55 ± 0.19^#^	0.89 ± 0.12**	1.86 ± 0.09**	0.71 ± 0.03**
AC	-0.08 ± -0.06	0.35 ± 0.02^#^	-0.12 ± -0.06**	0.10 ± 0.01**	0.01 ± 0.005**
CRR	0.92 ± 0.06	1.35 ± 0.04^#^	0.88 ± 0.06**	1.10 ± 0.08**	1.01 ± 0.07**
CAI	0.20 ± 0.04	0.85 ± 0.05^#^	0.06 ± 0.02**	0.47 ± 0.06**	0.15 ± 0.10**

The data are presented as means ± SD, *n* = 10. One-way ANOVA followed by the post hoc Tukey test. ^#^*p* < 0.01, compared to Control group. *p < 0.05 and **p < 0.01, compared to DIO group. Feno, fenofibrate; PFV, pomegranates fruits vinegar; CTFV, *C. tricuspidata* fruits vinegar; TC, total cholesterol (mg/dL); TG, triacylglycerol (mg/dL); HDL, high-density lipoprotein cholesterol (mg/dL); LDL, low-density lipoprotein cholesterol (mg/dL); VLDL, very low-density lipoprotein cholesterol (mg/dL); HTR, HDL/total cholesterol ratio; Glucose, TP (total protein), and albumin (mg/dL); Insulin (ng/mL); HOMA-IR, homeostasis model assessment-insulin resistance; AST, aspartate aminotransferase (U/L); ALT, alanine aminotransferase (U/L); Leptin (ng/mL); Adiponectin (μg/mL); AI, atherogenic index; AC, atherogenic coefficient; CRR, cardiac risk ratio; CAI, coronary artery index.

The indices including AI, AC, CRR, and CAI of mice were elevated after HFD feeding compared with the Control group, as shown in Table 7. The increased atherogenic index was strongly decreased in the CTFV group, and elevated values of the atherogenic coefficient, cardiac risk ratio, and coronary artery index were remarkably reduced in the Feno group. Differences in parameters such as glucose, total protein, albumin, leptin, and adiponectin were not significant in the CTFV group and other groups.

### CTFV efficacy involves alterations in AdipoR1, OBR, IRS1, and PTP1B signaling and its phosphorylation in liver of obese mice

**Figure 6:**
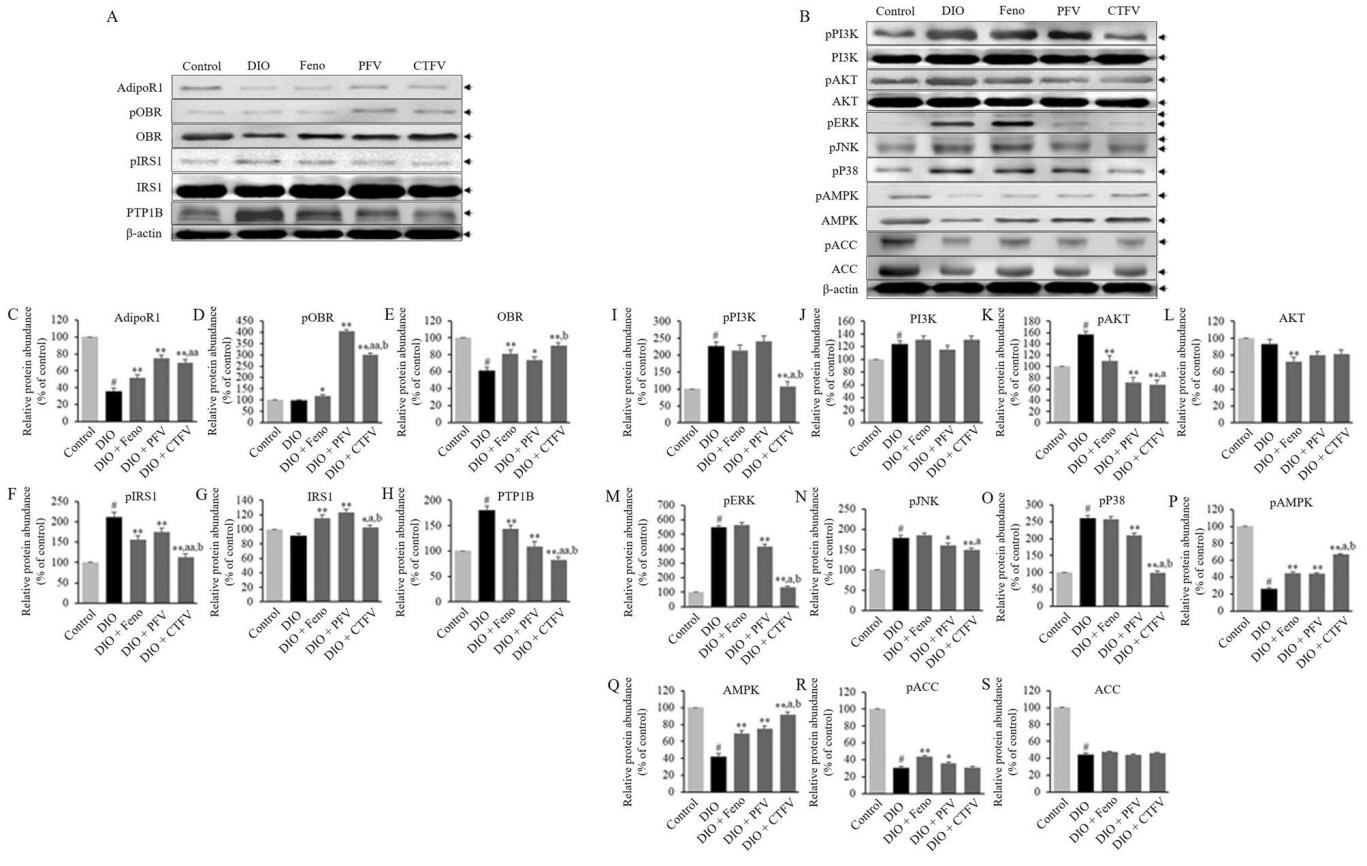
Effects of CTFV, PFV, and fenofibrate on AdipoR1, OBR, IRS1, and PTP1B (**A**), or PI3K, AKT, ERK, P38, AMPK, and ACC (**B**) expressions in liver from the obese mice. Each value is the mean ± SD of triplicate measurements. Protein expression was detected by western blotting. ^#^*p* < 0.01, compared with Control group, *p < 0.05 and **p < 0.01, compared with DIO group, ^a^*p* < 0.05 and ^aa^*p* < 0.01, compared with DIO + Feno group, ^b^*p* < 0.01, compared with DIO + PFV group. Control, non-induced normal group; DIO, diet-induced obesity model group; DIO + Feno, fenofibrate-treated DIO group, DIO + PFV, pomegranate fruit vinegar (PFV)-treated DIO group; DIO + CTFV, *C. tricuspidata* fruit vinegar (CTFV)-treated DIO group.

To investigate how CTFV altered the activation of receptor genes after HFD feeding, we evaluated the protein expression levels of adiponectin, leptin, and insulin-related genes. We found that HFD feeding downregulated AdipoR1 (Fig. 6C), and OBR (Fig. 6E), and upregulated the levels of PTP1B (Fig. 6H), which is an IR suppressor, and IRS1 phosphorylation (Fig. 6F) compared with the Control group, but no changes in the levels of pOBR (Fig. 6D), and IRS1 (Fig. 6G), as shown in Fig. 6A. Compared with the DIO group, CTFV administration upregulated AdipoR1, OBR, and IRS1 expression and OBR phosphorylation, whereas the expression of PTP1B and IRS1 phosphorylation was downregulated. In the Feno and PFV groups, AdipoR1, OBR, IRS1, and OBR phosphorylation were upregulated and IRS1 phosphorylation and PTP1B were downregulated compared with the DIO group. These results indicate that the regulation of AdipoR1, and OBR expression/activation through CTFV administration coincides with the reduction in HDF-induced initial activation of fatty acid oxidation and lipid synthesis in liver from obese mice.

### CTFV regulates HDF-induced activation/phosphorylation of PI3K/AKT/MAPKs and AMPK in liver of obese mice

We further examined whether CTFV administration contributed to attenuating HDF-induced activation and its underlying mechanisms. Our findings showed that HFD feeding activated the phosphorylation of PI3K/AKT (Fig. 6I–K) and MAPKs, including ERK (Fig. 6M), JNK (Fig. 6N), and P38 (Fig. 6O), and downregulated AMPK (Fig. 6Q), ACC (Fig. 6S), and their phosphorylation (Fig. 6P,R) compared with the Control group (Fig. 6B), but no changes in the level of AKT (Fig. 6L). In the CTFV group, the phosphorylation levels of PI3K/AKT and MAPKs were markedly decreased, and AMPK and its phosphorylation signaling were strongly activated compared with those of the DIO group, but differences in the pACC/ACC levels after CTFV administration were not significant. While Feno administration reduced AKT/pAKT levels and increased AMPK/pAMPK and pACC levels, PFV administration downregulated pAKT and MAPK activation and upregulated pAMPK/AMPK and pACC. These results demonstrate that CTFV administration regulates the underlying mechanisms after HDF-induced stimulation by attenuating the PI3K/AKT/MAPKs pathway and activating AMPK.

### CTFV controls HDF-induced protein abundance in energy/lipid metabolism in liver of obese mice

**Figure 7:**
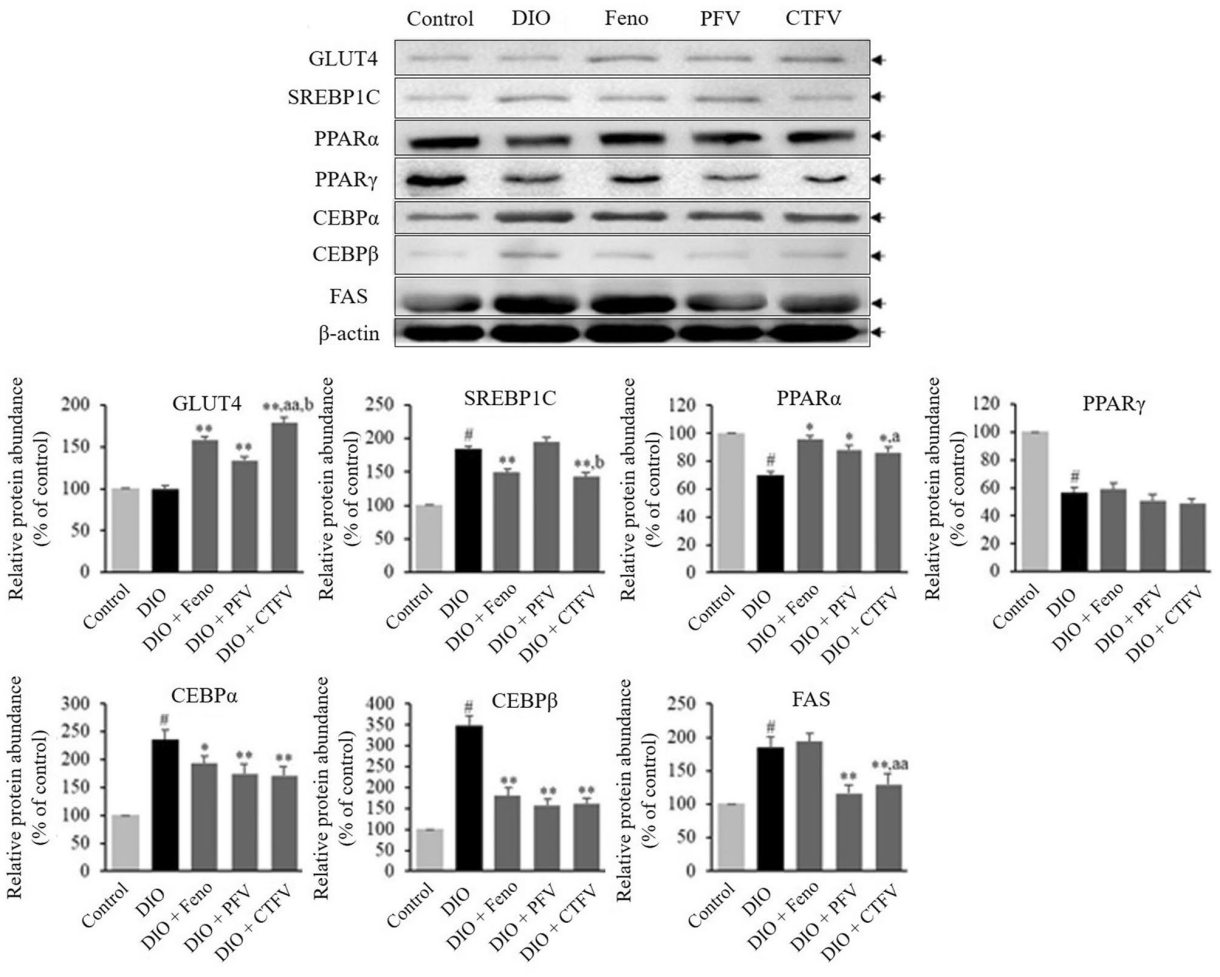
Effects of CTFV, PFV, and fenofibrate on GLUT4, SREBP1C, PPARα, PPARγ, CEBPα, CEBPβ, and FAS expressions in liver from the obese mice. Each value is the mean ± SD of triplicate measurements. Protein expression was detected by western blotting. ^#^*p* < 0.01, compared with Control group, *p < 0.01, compared with DIO group (A), ^a^*p* < 0.05 and ^aa^*p* < 0.01, compared with DIO + Feno group, ^b^*p* < 0.01, compared with DIO + PFV group. Control, non-induced normal group; DIO, diet-induced obesity model group; DIO + Feno, fenofibrate-treated DIO group, DIO + PFV, pomegranate fruit vinegar (PFV)-treated DIO group; DIO + CTFV, *C. tricuspidata* fruit vinegar (CTFV)-treated DIO group.

To investigate how CTFV controls the underlying molecular mechanisms involved in glucose transport, lipid synthesis, and fatty acid oxidation, the protein expression of GLUT4, SREBP1C, PPARα and γ, CEBPα and β, and FAS were determined in the liver of each group. As shown in Fig. 7, the DIO group presented reductions in PPARα and γ expression and elevations in SREBP1C, CEBPα and β, and FAS expression compared with those of the Control group. CTFV administration activated GLUT4 and PPARα expression and attenuated SREBP1C, CEBPα and β, and FAS expression. The upregulation in GLUT4 and PPARα expression and downregulation in CEBPα and β expression were observed in the Feno and PFV groups compared with the DIO group. Moreover, Feno administration inhibited SREBP1C expression, whereas PFV administration strongly inhibited FAS expression. Our findings demonstrate that CTFV administration contributed to the reduction in lipid and fat accumulation by downregulating SREBP1C, CEBPα, CEBPβ, and FAS or by activating GLUT4 and PPARα.

## Discussion

Vinegar has been used since about 300 BC and is a crucial component for food flavoring and preservation in European, Western, and Asian countries. While vinegar was once used as a simple seasoning, its use has expanded to health-related fields, and its various biological activities are actively undergoing study. Many bio-active ingredients, including amino acids, peptides, carbohydrates, organic acids, minerals, vitamins, and polyphenolics, that are effective for health care have been found in vinegar^43–45^. In particular, fruit vinegars produced using apples, grapes, pineapples, or berries contain various flavonoids and polyphenolics, and they are known to increase physiological abilities and improve in vitro and in vivo body fat accumulation and overweight states^43–47^. There are numerous articles regarding the anti-obesity effect of compounds detected from fruit vinegars, including caffeoylsophorose, chlorogenic acid, *p*-hydroxybenzoic acid, ferulic acid, syringic acid, and catechin^46,48–50^. For fruit vinegars, the anti-obesity abilities involving lipid or energy metabolism or glucose regulation are mostly due to acetic acid and other major substances. The previous studies demonstrated that acetic acid consumption decreased the blood glucose content and lipid synthesis via activating AMPK and reducing SREBP-1c or PPARγ levels among vinegar components^46,47,51,52^. Besides acetic acid as the major substance, caffeoylsophorose has an α-glucosidase-inhibitory function and a reductive effect on blood glucose^53^, while ferulic acid^54,55^ and syringic acid^56,57^ suppressed obesity-associated metabolism through reductions in lipogenesis (TG production), fat accumulation (body, hepatic), reactive oxygen species (ROS) accumulation, serum lipids (TC, TG, LDL), and serum inflammation (IL-6, TNF- α) or an increase in lipolysis (glycerol release).

In a previous study^58^, CTFV was examined to establish the conditions of the fermentation process using acetic acid isolated from traditional fermented food, and that article mainly considered the identification of bacterial strains in traditional foods, the chemical properties of vinegar, sensory evaluation, free radical scavenging, and α-glucosidase inhibition assays. The present study is the first to demonstrate the production and physicochemical characteristics of fruit vinegar using *C. tricuspidata* fruits, the detection of polyphenolic and parishin derivatives from CTFV, the metabolism-associated inhibitory effects of compounds detected in CTFV, and the anti-obesity effects of CTFV against a DIO mouse model.

Oxidative metabolisms are involved in the development of obesity. Bioactive adipokines produced from white adipose tissue are linked to the formation of ROS which induces oxidative stress by some mechanisms including the product of free radicals through excessive oxygen consumption, the peroxisomal or mitochondrial oxidation of fatty acids, and consumption of lipid or fat-rich diets^59,60^. Moreover, obesity and oxidative stress have been related to the development of the metabolic syndromes including insulin resistance, diabetes, systemic arterial hypertension, ischemic heart diseases, obstructive sleep apnea, asthma, gout, peripheral vascular disease, psychology problems, rheumatological problems, oncology problems, and liver failure^60^. The continuing symptoms of obesity gradually reduce antioxidant capabilities, resulting in reduced activity of antioxidant enzymes such as SOD^61^, CAT^61^ and GPx^62^ and eventually lead to obesity-related complications. In addition, the factors that are being applied as biomarkers for obesity and oxidative stress were reported by MDA^60^ and NO^63^. Increased production of superoxide and endothelial NO in obese patients may increase peroxynitrite level, reducing the usability of NO and inducing vasoconstriction in liver blood vessels^64^. Meanwhile, the decrease in body fat is well known to improve the oxidative parameters, increasing antioxidant capabilities, and these effects have been reported to be due to ingestion of key antioxidant substances such as vitamin C, vitamin E and flavonoids^60^.

The liver play a crucial role in the control of cholesterol, fatty acids, and some metabolites since it manages the reservation and elimination mechanisms through the AMPK/PI3K/AKT/MAPKs/PTP1B multi-pathway, including their underlying molecular signaling pathways. Three different major receptors involved in the energy-regulatory mechanism are AdipoR1, IR, and OBR, which react with their substrates to induce the activation of AMPK/PI3K/AKT/MAPKs and eventually contribute to fatty acid oxidation, lipid synthesis, and glucose uptake^65–71^. AMPK has been found as a mediator or key enzyme in energy metabolism, lipid homeostasis, and glucose uptake in liver and adipose tissue^72,73^. AMPK is activated/phosphorylated by fasting or starvation; furthermore, activated AMPK stimulates glucose uptake and lipid oxidation by inactivating 3-hydroxy-3-methylglutaryl CoA reductase and ACC as key metabolic enzymes, and it diminishes glycerolipid synthesis by decreasing the concentration of malonyl CoA^74,75^. The PI3K/AKT molecular signaling pathway involves several cellular functions via regulating growth factors in cellular development and organismal processes, including cell proliferation, protein synthesis, lipid metabolism, and glucose homeostasis^76^. The liver reacts closely with insulin through decreasing glucose levels, and the feeding state induces reductions in hepatic glucose production and increases in the synthesis of fatty acids and glycogen synthesis via PI3K/AKT/GLUT4 or PI3K/AKT/SREBP-1c signaling^77–79^. SREBPs are transcription factors that control gene expression in the biosynthesis of phospholipids, triglycerides, fatty acids, and cholesterol; particularly, SREBP-1c mediates genes expression involved in triacylglycerol accumulation and synthesis, including ACC and FAS^80,81^. SREBP-1c overexpression causes intensive hepatic lipogenesis. AKT modulates the signaling of mammalian target of rapamycin complex 1-p70 ribosomal protein S6 kinase 1 (mTORC1-S6K1) to mediate SREBP-1c expression in the liver and inhibits the expression of insulin-induced gene (INSIG) 2a, which encodes the SREBP-1c inhibitor, via an mTORC1-independent mechanism^78^. MAPKs such as ERK, JNK, and P38 are involved in the essential processes for cell differentiation and proliferation, are known to contribute to adipocyte differentiation by regulating C/EBPα and PPARγ expression, and are phosphorylated through IR and IRS1/2, which are activated by increasing glucose and insulin as adipogenic stimuli^82,83^. PTP-1B is a negative regulator of IR signal transduction and a potential target for the treatment of metabolic diseases, including type 2 diabetes and obesity^84^. Mice lacking PTP1B are hypersensitive to leptin resistance, obesity, and insulin level^85^. The signaling pathways induced by adipocytokines and adipogenic stimuli eventually contribute to controlling the signal transductions of PTP1B, ACC, SREBP, GLUT, PPAR, C/EBP, and FAS to regulate insulin or leptin signaling, fatty acid oxidation, glucose uptake, lipid synthesis, and adipose differentiation.

In the present study, HFD feeding for 50 days caused phenotypic changes including hyperlipidemia, hepatic steatosis, and increased adiposity and liver mass similar to those found in previous studies^36,86–88^. HFD feeding increased the body weight gain by 2.11-fold, body fats (epididymal, perirenal, and mesenteric) by 3.15–4.40-fold, hepatic fat mass by 1.34-fold, and adipocyte size by 2.01-fold compared to mice fed a normal diet, and these changes might be correlated with hypercholesterolemia, hypertriglyceridemia, hepatomegaly, insulin resistance, and cardiovascular-related risk indices. Our findings reveal that HFD feeding elevated TC, TG, LDL, insulin resistance, liver mass, and cardiovascular-related risk indices (including AI, AC, CRR, CAI) by 1.55, 2.01, 4.59, 5.23, 1.67, and 1.47–4.25-fold, respectively, compared to the normal mice. Moreover, liver function tests for biochemical abnormalities, a rise in leptin level, reduction in adiponectin level, changes in obesity-associated metabolizing enzyme activities, and oxidative damages were observed in liver and serum from the obese mice model. According to the present findings, CTFV surpassed PFV (functional foods to help reduce body fat) and fenofibrate (a lipid-lowering drug) in reducing body weight gain, hepatic fat or mass, body fats, adipocyte size, serum or liver triacylglycerol, leptin, the atherogenic index, and nitrite, as well as in increasing adiponectin and SOD. The possible mechanisms of the strong anti-obesity ability of CTFV might be due to the presence of metabolites and substances such as chlorogenic acid, caffeic acid, rutin, gastrodin, 4-hydroxybenzoic acid, parishin A, and acetic acid in CTFV, which were known to have anti-oxidative^32,89,90^, anti-obesity^11,18,90–95^, cardio-protective^89,95^, and anti-inflammatory effects^11,95^.

Liver steatosis and nonalcoholic fatty liver are the most common chronic liver disease. In previous studies^96,97^, fatty liver is commonly caused by high-carbohydrate or high-fat ingestion, hormonal manipulation, and dietary methionine restriction. Low steady-state TG levels of liver on physiological condition were maintained by balancing between accumulation and disposal. Under conditions of increased nutrition and insulin resistance, the balance is destructed by various factors that increase the concentration of TGs and VLDLs in the liver, which in turn results in liver steatosis. Our findings show HFD-induced increased levels of TC, TH, LDL, and VLDL resulted in hepatic steatosis and increase in hepatic TG level. Moreover, the lipid accumulation in the liver can be caused by four different metabolic reactions: decreased oxidation of hepatic fatty acids, increase in transference of fatty acids (TG in adipose cells, dietary lipids, hepatic de novo lipogenesis) to hepatocytes, inadequate secretion of neutral fats in VLDL, and increased synthesis of TG^98^.

Recently, anti-obesity studies have concentrated on testing for reductions in the activities of obesity-associated metabolizing enzymes, because inhibiting these enzymatic activities in energy/carbohydrate/lipid metabolisms can reduce metabolite usage and absorption by activating thermogenesis and lipolysis and by inhibiting fatty acid formation and digestive activities^28,30,99,100^. Here, our findings demonstrated that CTFV and its phytophenolics including chlorogenic acid, caffeic acid, rutin, gastrodin, 4-hydroxybenzoic acid, and parishin A inhibited the obesity-related metabolizing enzymes of lipase, β-glucosidase, α-amylase, citrate synthase, and alkaline phosphatase in vitro, suggesting that CTFV treatment reduced lipid-hydrolyzing activity, triacylglycerol hydrolysis^101^, glycogenolysis^102^, glucose absorption^33^, the initial material concentration for fatty acid synthesis^30^, and lipid absorption or transport^103^. Although CTFV induced mean decreases in enzymatic activities in vivo, there were some differences compared with the effects in vitro. CTFV administration showed significant inhibitory effects in vivo against the activities of pancreatic lipase and alkaline phosphatase in serum and α-amylase and lipoprotein lipase in liver from the HFD-induced obese mice model. The difference between the in vitro or in vivo effects is not understood; however, these results suggest that trace amounts of phytophenolics present in CTFV are transported to the liver through the bloodstream; then, they partially influence enzyme activity in the blood and liver of the obese mice^104^. In addition, the immunoblotting results demonstrated that the effects of CTFV contributed AMPK activation, and reduction of initial activation of fatty acid oxidation and lipid synthesis, phosphorylation stimulation, and lipid or fat accumulation by influencing hepatic AdipoR1, OBR, IRS1, PTP1B, PI3K/AKT/MAPKs, AMPK, and various signaling transductions.

In conclusion, the present study demonstrated that CTFV inhibited the HFD-induced increases in body weight, feed efficiency, body fat mass, adipose cell size, and serum or liver lipids by regulating signal transduction mechanisms related to adipogenesis, fatty acid oxidation, and glucose transport and by mediating obesity-associated enzymatic activities and the antioxidant ability in liver or serum. Therefore, we suggest that CTFV might be beneficial for improving HFD-induced obesity and for developing functional food ingredients with an anti-obesity ability.

## Supplementary information

Supplementary Information.
