# Supplementary material for: Short-term Cudrania tricuspidata fruit vinegar administration attenuates obesity in high-fat diet-fed mice by improving fat accumulation and metabolic parameters
Source: Sci Rep. 2020 Dec 3;10:21102. doi: 10.1038/s41598-020-78166-9 (PMC7712837; doi:10.1038/s41598-020-78166-9)

**Supplementary Information for**

**Short-term *Cudrania tricuspidata* fruit vinegar administration attenuates obesity in high-fat diet-fed mice by improving fat accumulation and metabolic parameters**

Jun-Hui Choi^1^, Myung-Kon Kim^2^, Soo-Hwan Yeo^3^, Seung Kim^1*^

^1^Department of Food Science and Biotechnology, Gwangju University, Gwangju, 503-703, Republic of Korea

^2^Department of Food Science and Technology, Chonbuk National University, Iksan 570-752, Republic of Korea

^3^Department of Agro-food Resource, National Academy of Agricultural Science, RDA, Suwon 441-853, Republic of Korea

Current file content:

• Figure S1. Effects of CTFV, PFV, and fenofibrate on AdipoR1 protein expression in liver from the obese mice.

• Figure S2. Effects of CTFV, PFV, and fenofibrate on phosphorylated OBR protein expression in liver from the obese mice.

• Figure S3. Effects of CTFV, PFV, and fenofibrate on OBR protein expression in liver from the obese mice.

• Figure S4. Effects of CTFV, PFV, and fenofibrate on phosphorylated IRS1 protein expression in liver from the obese mice.

• Figure S5. Effects of CTFV, PFV, and fenofibrate on IRS1 protein expression in liver from the obese mice.

• Figure S6. Effects of CTFV, PFV, and fenofibrate on PTP1B protein expression in liver from the obese mice.

• Figure S7. The loading control blot in liver from the obese mice.

• Figure S8. Effects of CTFV, PFV, and fenofibrate on phosphorylated PI3K protein expression in liver from the obese mice.

• Figure S9. Effects of CTFV, PFV, and fenofibrate on PI3K protein expression in liver from the obese mice.

• Figure S10. Effects of CTFV, PFV, and fenofibrate on phosphorylated AKT protein expression in liver from the obese mice.

• Figure S11. Effects of CTFV, PFV, and fenofibrate on AKT protein expression in liver from the obese mice.

• Figure S12. Effects of CTFV, PFV, and fenofibrate on phosphorylated ERK protein expression in liver from the obese mice.

• Figure S13. Effects of CTFV, PFV, and fenofibrate on phosphorylated JNK protein expression in liver from the obese mice.

• Figure S14. Effects of CTFV, PFV, and fenofibrate on phosphorylated P38 protein expression in liver from the obese mice.

• Figure S15. Effects of CTFV, PFV, and fenofibrate on phosphorylated AMPK protein expression in liver from the obese mice.

• Figure S16. Effects of CTFV, PFV, and fenofibrate on AMPK protein expression in liver from the obese mice.

• Figure S17. Effects of CTFV, PFV, and fenofibrate on phosphorylated ACC protein expression in liver from the obese mice.

• Figure S18. Effects of CTFV, PFV, and fenofibrate on ACC protein expression in liver from the obese mice.

• Figure S19. The loading control blot in liver from the obese mice.

• Figure S20. Effects of CTFV, PFV, and fenofibrate on GLUT4 protein expression in liver from the obese mice.

• Figure S21. Effects of CTFV, PFV, and fenofibrate on phosphorylated SREBP1C protein expression in liver from the obese mice.

• Figure S22. Effects of CTFV, PFV, and fenofibrate on PPARα protein expression in liver from the obese mice.

• Figure S23. Effects of CTFV, PFV, and fenofibrate on PPARγ protein expression in liver from the obese mice.

• Figure S24. Effects of CTFV, PFV, and fenofibrate on CEBPα protein expression in liver from the obese mice.

• Figure S25. Effects of CTFV, PFV, and fenofibrate on CEBPβ protein expression in liver from the obese mice.

• Figure S26. Effects of CTFV, PFV, and fenofibrate on FAS protein expression in liver from the obese mice.

• Figure S27. The loading control blot in liver from the obese mice.

Figure S1


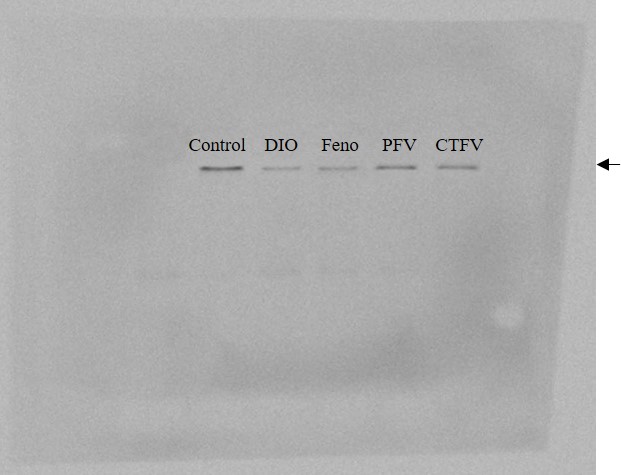


Figure S2


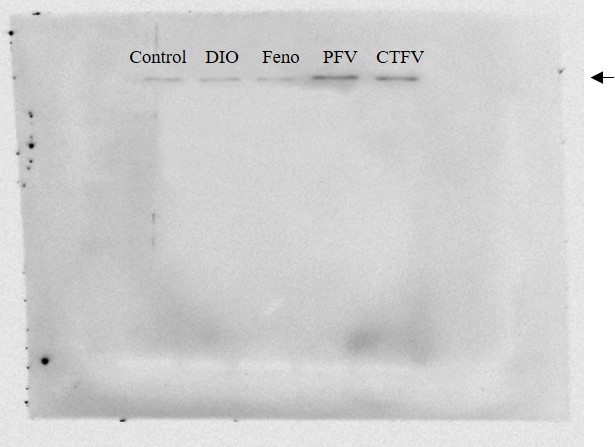


Figure S3


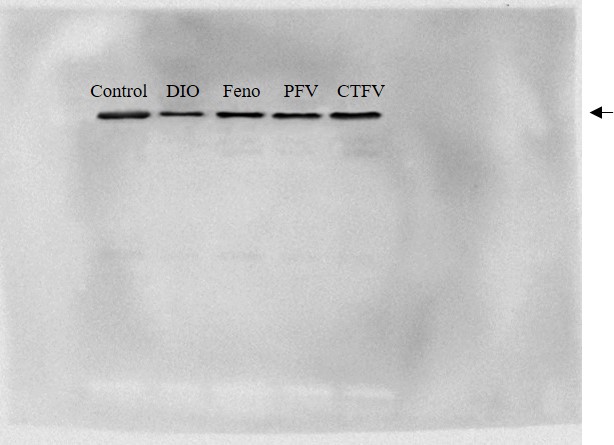


Figure S4


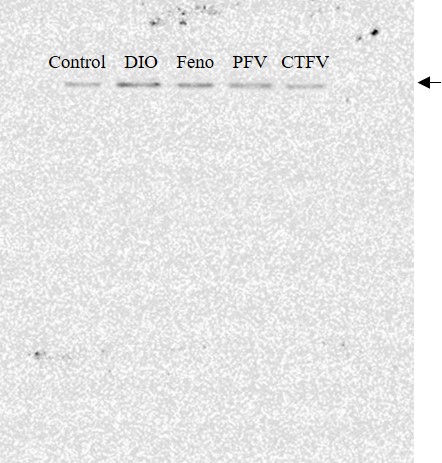


Figure S5


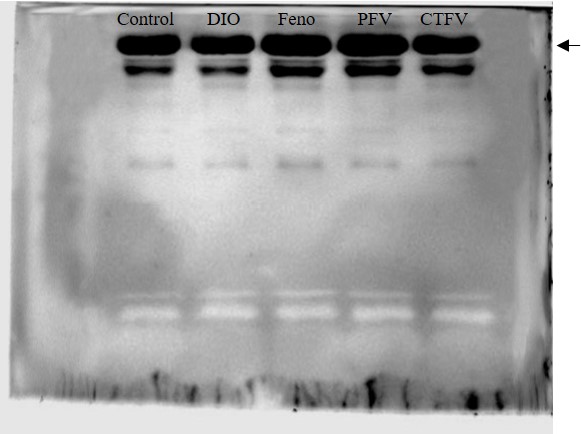


Figure S6


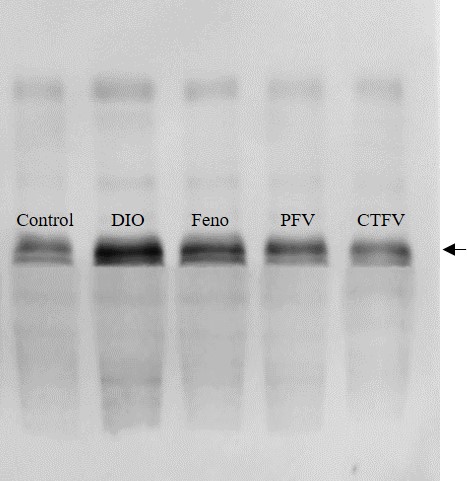


Figure S7


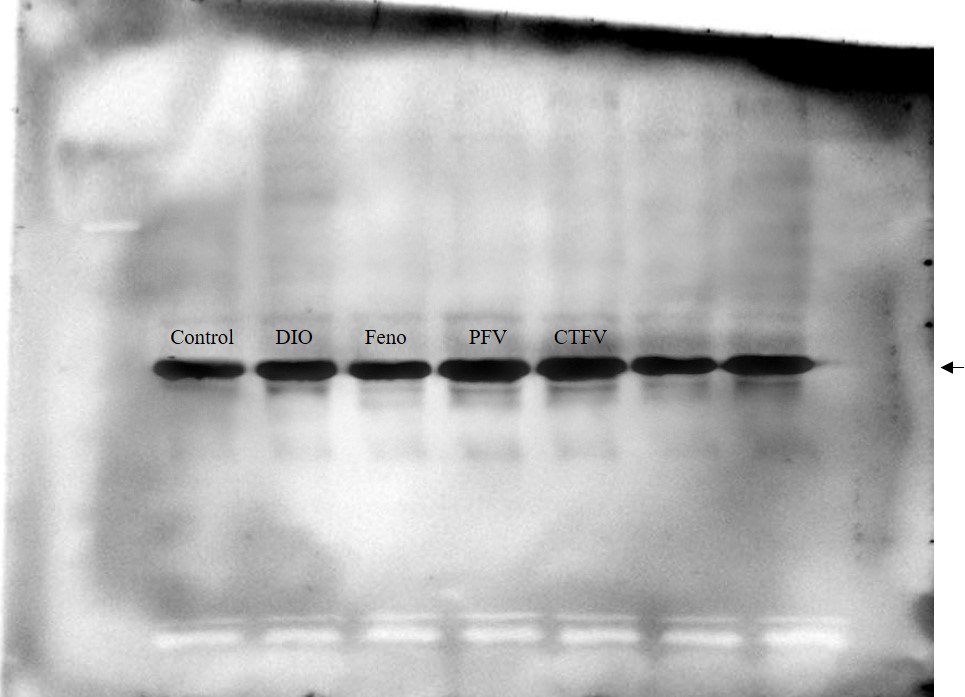


Figure S8


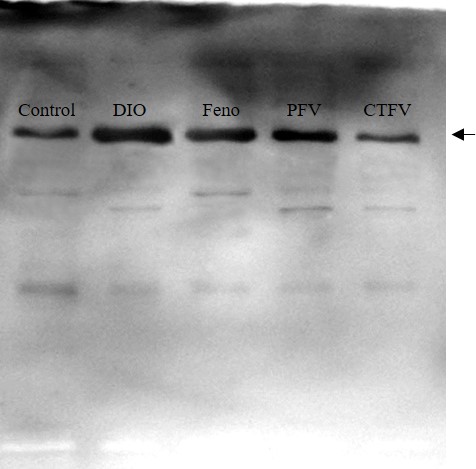


Figure S9


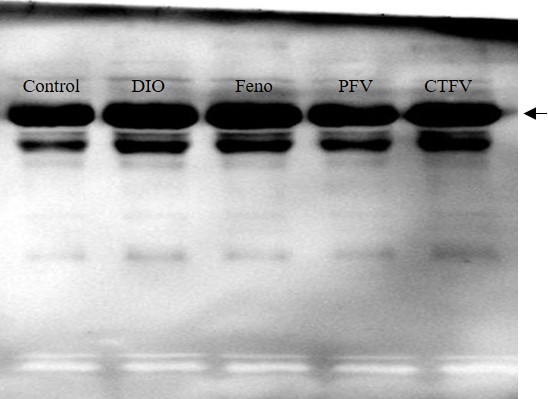


Figure S10


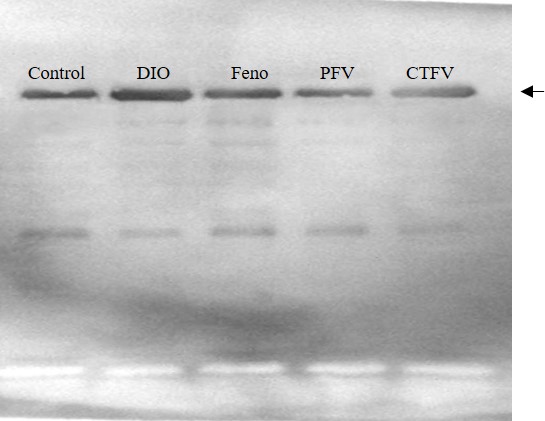


Figure S11


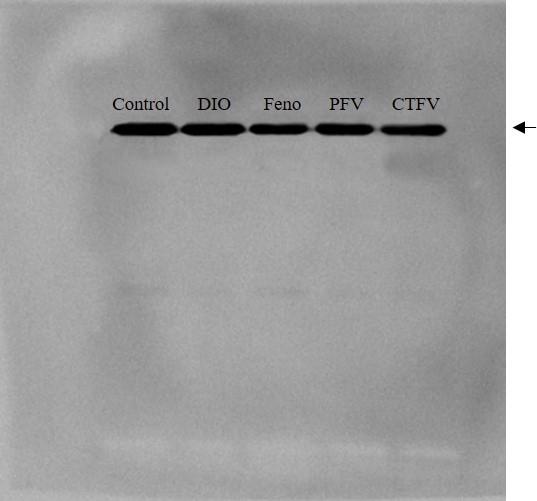


Figure S12


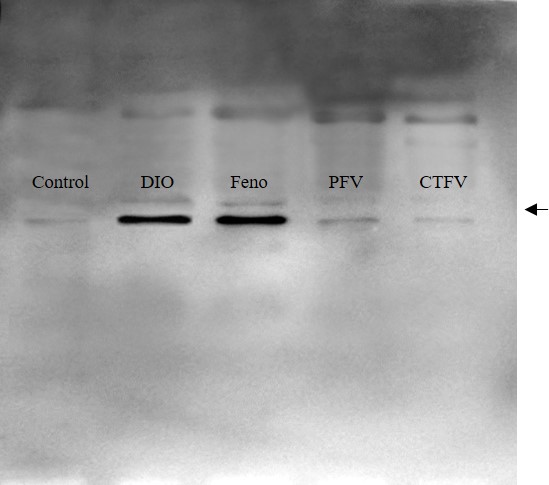


Figure S13


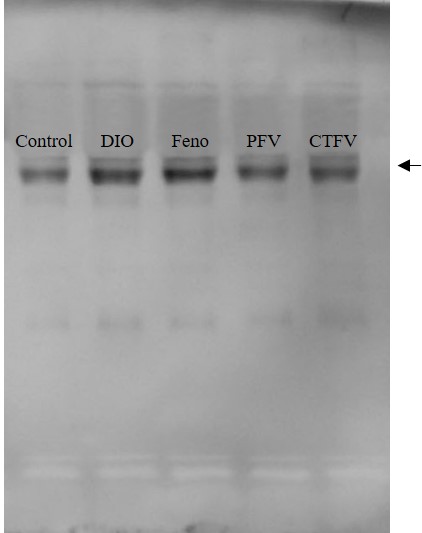


Figure S14


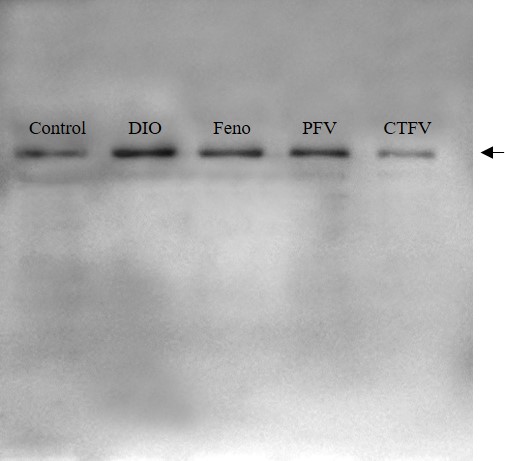


Figure S15


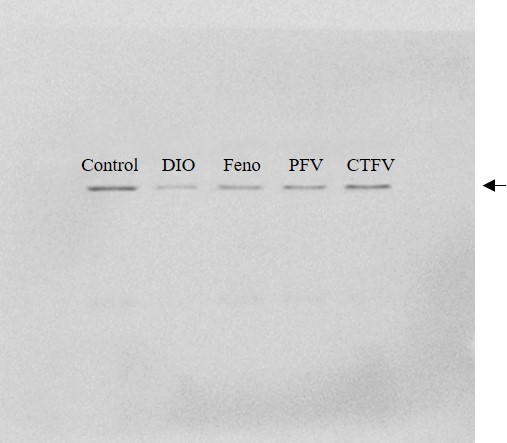


Figure S16


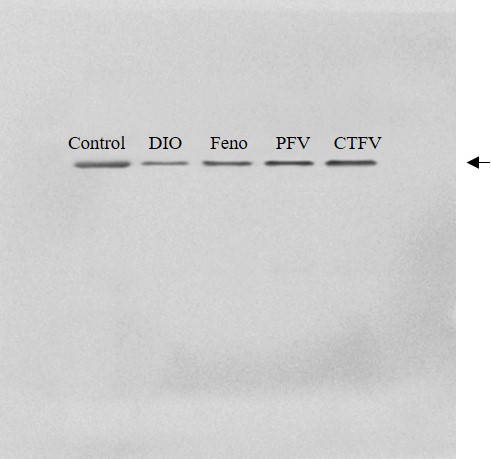


Figure S17


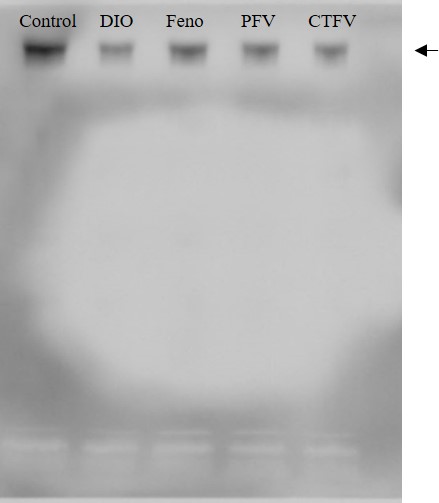


Figure S18


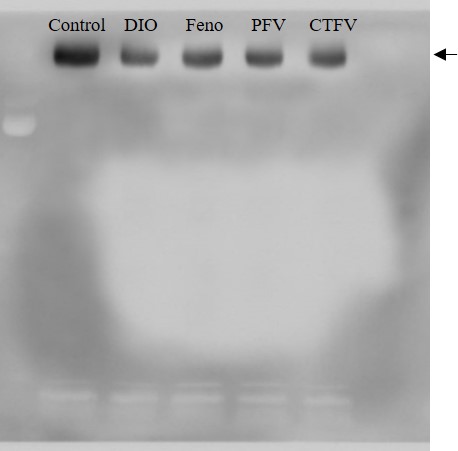


Figure S19


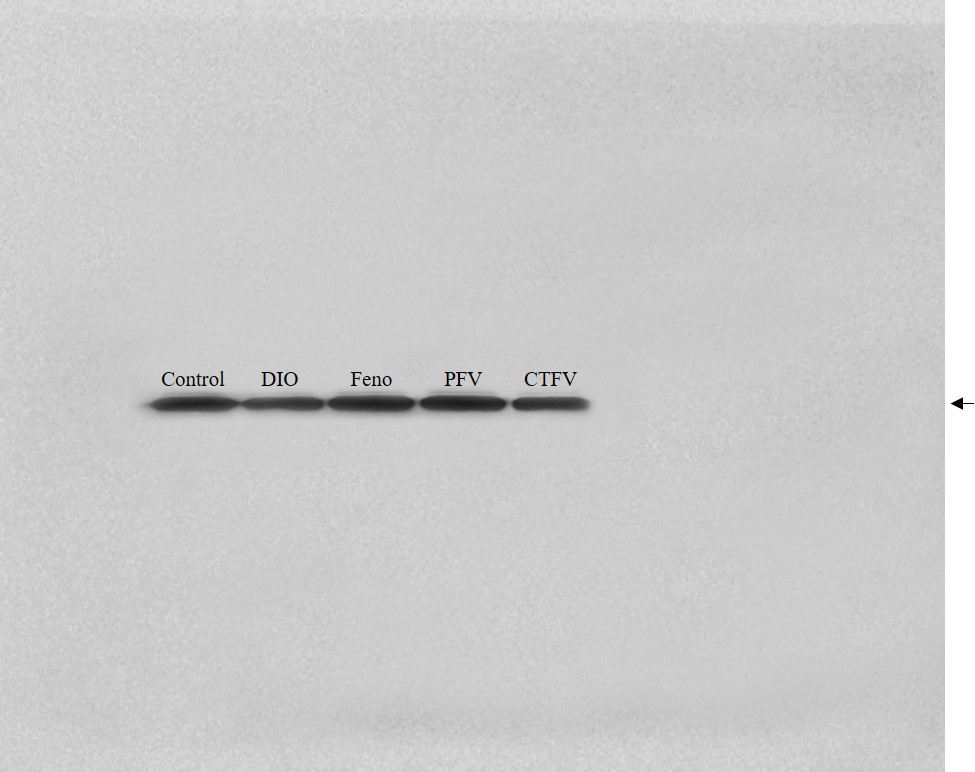


Figure S20


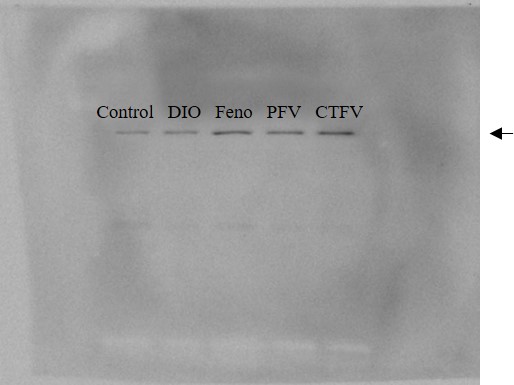


Figure S21


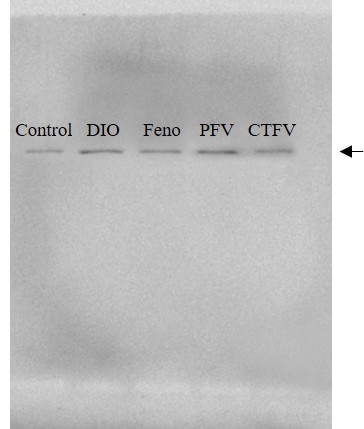


Figure S22


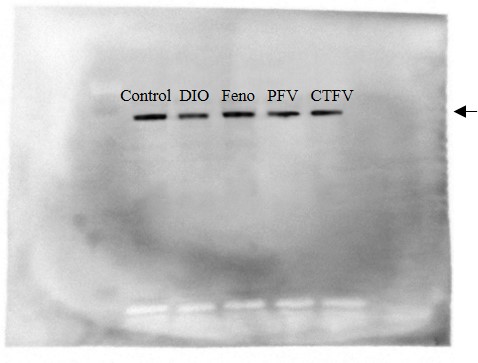


Figure S23


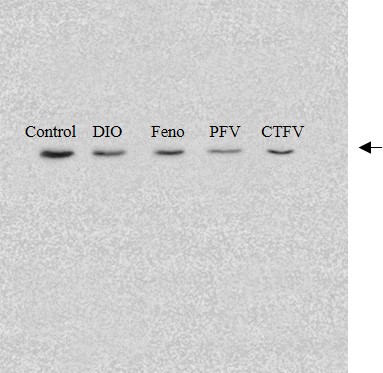


Figure S24


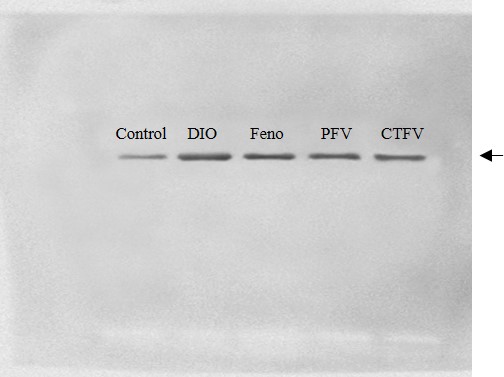


Figure S25


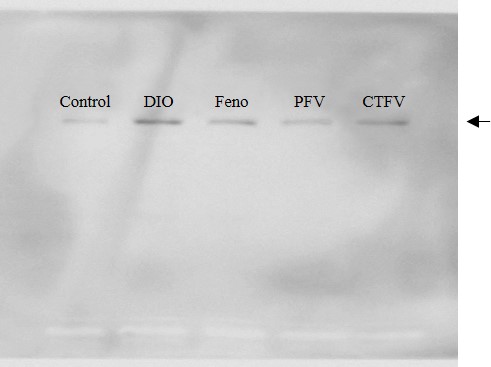


Figure S26


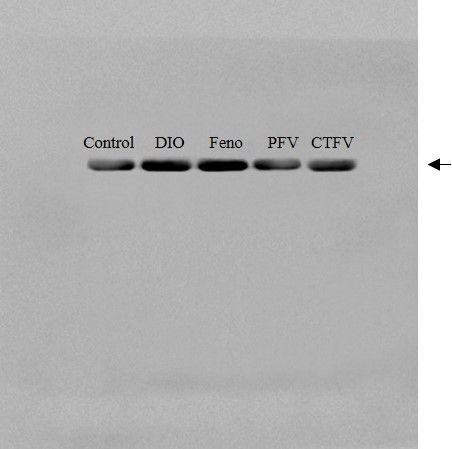


Figure S27


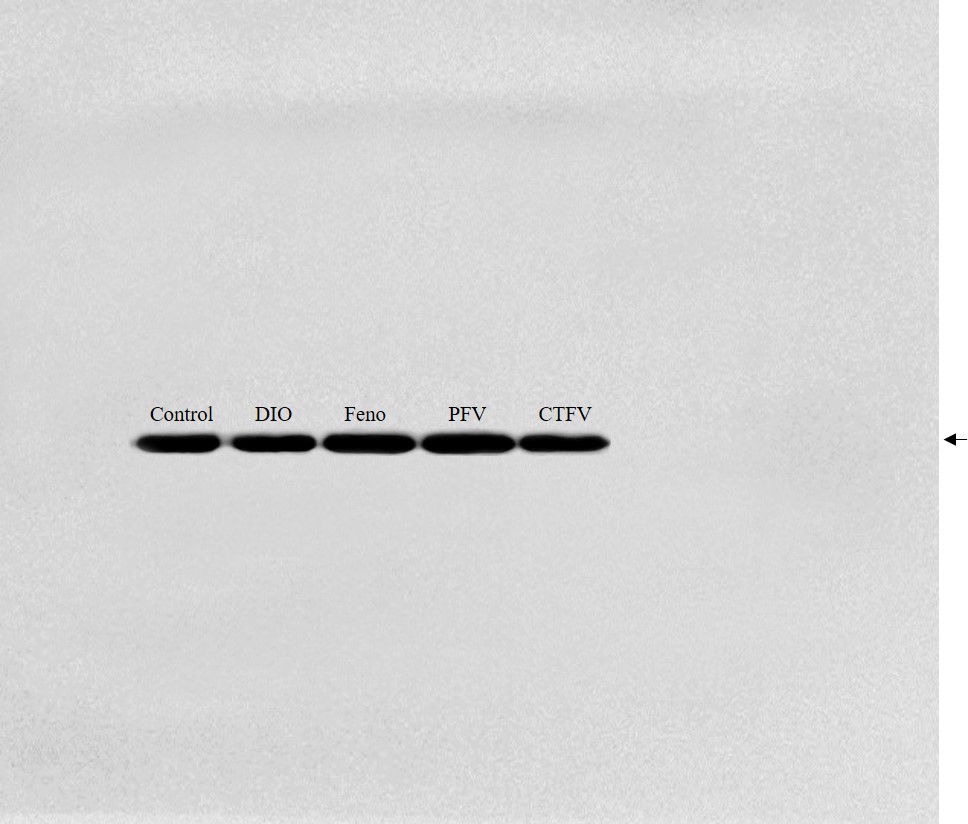

Supplement: Supplementary file 1 — Supplementary Information. [file 41598_2020_78166_MOESM1_ESM.docx]
